# The circuitry regulation of associative learning: dissociated and integrated function of the perirhinal cortex and hippocampus

**DOI:** 10.3389/fncir.2026.1789080

**Published:** 2026-03-17

**Authors:** Jingyi Zhang, Xiaohui Zhang

**Affiliations:** State Key Laboratory of Cognitive Neuroscience and Learning, IDG/McGovern Institute for Brain Research, Beijing Normal University, Beijing, China

**Keywords:** associative learning, episodic memory, hippocampus, multisensory integration, perirhinal cortex

## Abstract

The formation of associations, which involves binding disparate pieces of information, is fundamental to constructing episodic memory. This process primarily relies on the neural circuitry within the medial temporal lobe, specifically the hippocampal-parahippocampal network. Within this network, the perirhinal cortex (PER) and the hippocampus (HPC) are recognized as essential components for associative processing. While the traditional dual-pathway model depicts a hierarchically organized, sequential transmission of information along the medial temporal lobe, recent anatomical and functional studies reveal that the PER and HPC are embedded within a far more extensive and complex multi-pathway connectivity architecture. These connections enable parallel and dynamic interactions between PER, HPC, and other medial temporal lobe structures, supporting flexible modes of information processing and integration essential for associative learning. This review systematically re-evaluates the roles of the PER and HPC in associative learning. We begin by advancing the view that the PER acts not as a passive sensory gateway, but as an associative hub for multimodal association formation, whose special local inhibition provides the computational foundation for integrating complex information of both object features, and spatiotemporal context or affective valence. Building on this perspective, we then synthesize evidence on the dynamic interactions between the PER and HPC, encompassing findings from extensive anatomical and electrophysiological studies. Finally, we focus on the HPC, elucidating how it precisely coordinates information from the PER and other regions, with a particular emphasis on the critical regulatory roles played by inhibitory neurons in this integrative process. The reciprocal neuronal connections, coherent neuronal oscillatory activities and shared neuromodulation in the PER-HPC circuit facilitate the integration of associative learning.

## 1 Introduction

Episodic memory is a crucial cognitive function that supports daily learning and normal life in humans. The formation of episodic memory is inseparable from associative learning-the process of binding disparate elements (such as objects, contexts, and emotions) into a coherent representation. Early studies demonstrated that damage to the hippocampus leads to memory deficits, impairing the formation of new memories and severely affecting an individual’s social functioning (Scoville and Milner, 1957; Pishdadian et al., 2024). As the research progressed, anatomical evidence has further revealed that episodic memory relies on the coordinated activity of the hippocampus and numerous surrounding brain regions, including the fornix, amygdala, parahippocampal regions, temporal olfactory cortex, and prefrontal cortex (Brown and Aggleton, 2001). Notably, the hippocampus, together with subregions of the parahippocampal region such as the entorhinal, perirhinal, postrhinal, pre-subiculum, and para-subiculum cortices, forms the hippocampal-parahippocampal network, which is considered a central hub for information integration and association formation (Witter et al., 2006; Burwell, 2024).

Building on this foundation, researchers have sought to elucidate how sensory information is processed through these regions to ultimately form memories. Inspired by hierarchical models of the visual perception pathway, the field proposed the classic “dual-stream hierarchical model” of episodic memory formation (Aggleton and Brown, 2005; Burwell, 2006; Furtak et al., 2007). This model posits that mnemonic information is processed sequentially across different brain areas, ultimately converging in the HPC to form coherent and integrated episodic representations. Within this framework, the item information is mediated by the PER and relayed forward to the lateral entorhinal cortex (LEC) before being further transmitted to the HPC. On the other hand, the processing of spatial information is predominantly mediated by the postrhinal cortex (POR) and the medial entorhinal cortex (MEC). Although these multiple brain regions cooperate in the formation of episodic memory, research focus has consistently centered on key integration hubs: the PER and HPC. Specifically, the PER is regarded as a critical association hub which receives inputs from high-order association cortex, primarily integrates complex object information from multisensory modalities such as olfaction, vision, and touch. In parallel, the HPC receives and converges two information streams, ultimately constructing coherent episodic memories that encompass scenes and spatiotemporal contexts. This theory has been substantiated by extensive neuroanatomical tracing, brain lesion studies, and electrophysiological experiments (Miyashita, 2019), and the coordination between the PER and HPC promotes associative formation (Jo and Lee, 2010; Yang and Naya, 2023).

In recent years, the widespread application of techniques such as optogenetics, *in vivo* multichannel electrophysiology, calcium imaging, and circuit-specific tracing has enabled the detection of dynamic, high-temporal-resolution neural activity changes across multiple brain regions during memory processes. These advances have led to a deeper understanding of memory processing, revealing the limitations of earlier, relatively static models based on functional segregation and unidirectional hierarchy. Based on these new evidence, Burke et al. (2018) proposed a process-based dual-stream hierarchical model that incorporates coarse/detail networks based on distinct connection patterns between the PER and HPC. The coarse network transmits “gist-like” information through an indirect pathway, whereas the detailed network conveys information through a direct pathway. Importantly, this refined model perspective extends to the microcircuit level. In addition, studies have shown that local inhibitory neurons (INs) play critical and specific regulatory roles in both the PER or HPC, respectively—with the diversity of INs types, origins, morphologies, and electrical properties being particularly well characterized in HPC CA1 (Pelkey et al., 2017; Topolnik and Tamboli, 2022).

Collectively, this review summarizes theoretical and experimental evidence regarding the cellular composition within the PER, circuit architecture and information flow between the PER and HPC, and the regulatory roles of inhibitory neurons in CA1 on information integration. It delineates how these two structures collaborate to construct, consolidate, and retrieve episodic memories.

## 2 How PER forms associations

### 2.1 Local inhibition is the precondition of association

As a gateway for sensory information entering the hippocampal system, the perirhinal cortex operates not merely as a relay station, but as a functional hub that dynamically filters inputs via intrinsic regulatory mechanisms such as membrane resonance and inhibitory circuits. Consistent with a common motif across neocortical regions, the perirhinal cortex utilizes robust local inhibition to achieve sparse neural representations (Barinka et al., 2012; Salaj et al., 2021; Schneider-Mizell et al., 2025). This stands in contrast to the dentate gyrus, which primarily employs pattern separation to orthogonalize inputs (Aimone et al., 2010). The intrinsically strong inhibition within PER ensures that only selected information with associative relevance undergoes sparse encoding, thereby creating a preconditioned substrate for subsequent cross-modal and contextual integration within the hippocampus (de Curtis and Paré, 2004; Willems et al., 2018; Kajiwara and Tominaga, 2021; Salaj et al., 2021).

Although local inhibitory microcircuits are widely organized across the neocortex, the potency of inhibition within PER is particularly striking, underpinning its ability for highly selective information filtering. Early experimental evidence revealed a pivotal phenomenon: despite anatomical studies demonstrating dense reciprocal connections between PER and EC via dye tracing (Burwell et al., 1995; Burwell and Amaral, 1998a, b; de Villers-Sidani et al., 2004; Furtak et al., 2007), neurophysiological recordings indicated low functional synchrony and inefficient information transfer between these regions (Biella et al., 2002; de Curtis and Paré, 2004). Further investigations highlighted that the EC, as a downstream areas from the PER and the gate of sensory information into HPC, responded sparsely to inputs from neocortical areas (Pelletier et al., 2004). Collectively, these evidence demonstrated that PER exhibits low efficacy in excitatory signal transmission to EC, implying its potential role as a critical information filter between the neocortex and EC. A plausible mechanism is that inputs concurrently excite pyramidal cells (PCs) and more potently activate local GABAergic inhibitory neurons, thereby filtering and restricting excitatory signal propagation through robust feedforward inhibition (Martina et al., 2001; Pinto et al., 2006; Willems et al., 2018). This provides direct evidence for the microcircuit mechanism of coordinated excitation and inhibition, demonstrating that neocortical inputs can promptly initiate both fast and sustained inhibitory feedback to block signals within the local PER network.

The inhibitory regulation within the PER might be distinguished from other primary sensory cortices because of the difference in dominant cell types. The parvalbumin (PV)-dominant pattern is existed in primary sensory cortices while it’s the calretinin (CR)-dominant pattern within the PER. We propose that the high density of CR neurons in the PER may serve as a critical gating switch within its local inhibitory microcircuitry. Cortical inhibition is generally mediated by PV neurons (primarily via GABA_*A*_ receptors) and somatostatin (SOM) neurons (partly via GABA_*B*_ receptors) (Shen et al., 2022; Huang et al., 2023). In the neocortical hierarchy, primary sensory and motor cortices are dominated by PV neurons, which provide fast and powerful feedback inhibition to ensure synchronous and precise neuronal firing, essential for encoding rapidly changing sensory stimuli and fine motor coordination (Wu et al., 2022). In comparison, higher-order associative cortices, including the PER and prefrontal cortex (PFC), show a CR-dominant trend. In adult mice, CR and vasoactive intestinal peptide (VIP) neurons are highly co-localized and functionally analogous (Camillo et al., 2018; Medalla et al., 2023). These CR neurons are densely localized in superficial layers (II/III), the key site for integrating multimodal sensory inputs in PER. Their axon terminals form dense plexuses, enabling individual CR neurons to simultaneously modulate excitability across broad regions (Medalla et al., 2023). Functionally, CR neurons disinhibit PCs by suppressing other interneurons, acting as a network “starter signal” to transiently enhance PC responsiveness to novel stimuli (Guet-McCreight et al., 2020), similar in the HPC (Francavilla et al., 2015). Concurrently, synchronized PC activation recruits feedback inhibition, for instance, via SOM neurons, which induce slow, persistent suppression potentially mediated by GABA_*B*_ receptors, to form an “inhibitory wall” to prevent network hyperexcitability. Although the inhibitory role of SOM neurons is well-documented (Liguz-Lecznar et al., 2016), but their function particularly in PER are still unclear. Whatever, the specific contribution of GABAergic receptors to this process has already been confirmed (Ziakopoulos et al., 2000; Kotak et al., 2017). In summary, the synergistic interaction between CR and other interneurons enable dynamic and precise regulation of excitation-inhibition balance in PER.

Importantly, CR neurons are modulated by top-down inputs (e.g., from prefrontal areas), allowing behavioral context, attention, or prior experience to dynamically regulate information gain through disinhibition (Obermayer et al., 2019; Zhu et al., 2025). When these top-down inputs arrive, may elicit more CR neurons in PER which disinhibit PCs, thereby transient open the “inhibitory wall” to permit excitatory signal transmission.

Another study demonstrates that the coupling between neuronal population firing and individual neuron activity within the perirhinal cortex is remarkably low, indicating an absence of highly synchronized firing patterns and indirectly supporting the existence of robust local inhibitory regulation (Dorman et al., 2023). The index of spike-based coupling is calculated by cross-correlation of single neurons and populations, which reflects their synchronization and coordination at different lags. Evidence comes from this research, which quantified the coupling based on firing rates between individual neurons and the larger networks in the region, showing that the degree of synchronization within PER is significantly low. Notably, neurons with spatial selectivity displayed even further reduced intra-regional coupling, and this coupling was inversely correlated with information specificity. This result reveals that neurons that encode more specific information exhibit much weaker coupling with population firing pattern in the PER, suggesting that local strong inhibition may underlie the formation of highly specific representations by reducing inter-neuronal synchronization. Additionally, the coupling between PER neurons and other regions such as primary visual cortex, the barrel field of primary somatosensory cortex and CA1 is also low enough, indicating that information encoding in PER neurons are not widely broadcast to other regions. Collectively, these findings characterize PER as a low-coupling region that participates less in broad synchronous population activity and may support more selective or sparse information processing, rather than broad synchronous information transmission.

**FIGURE 1:**
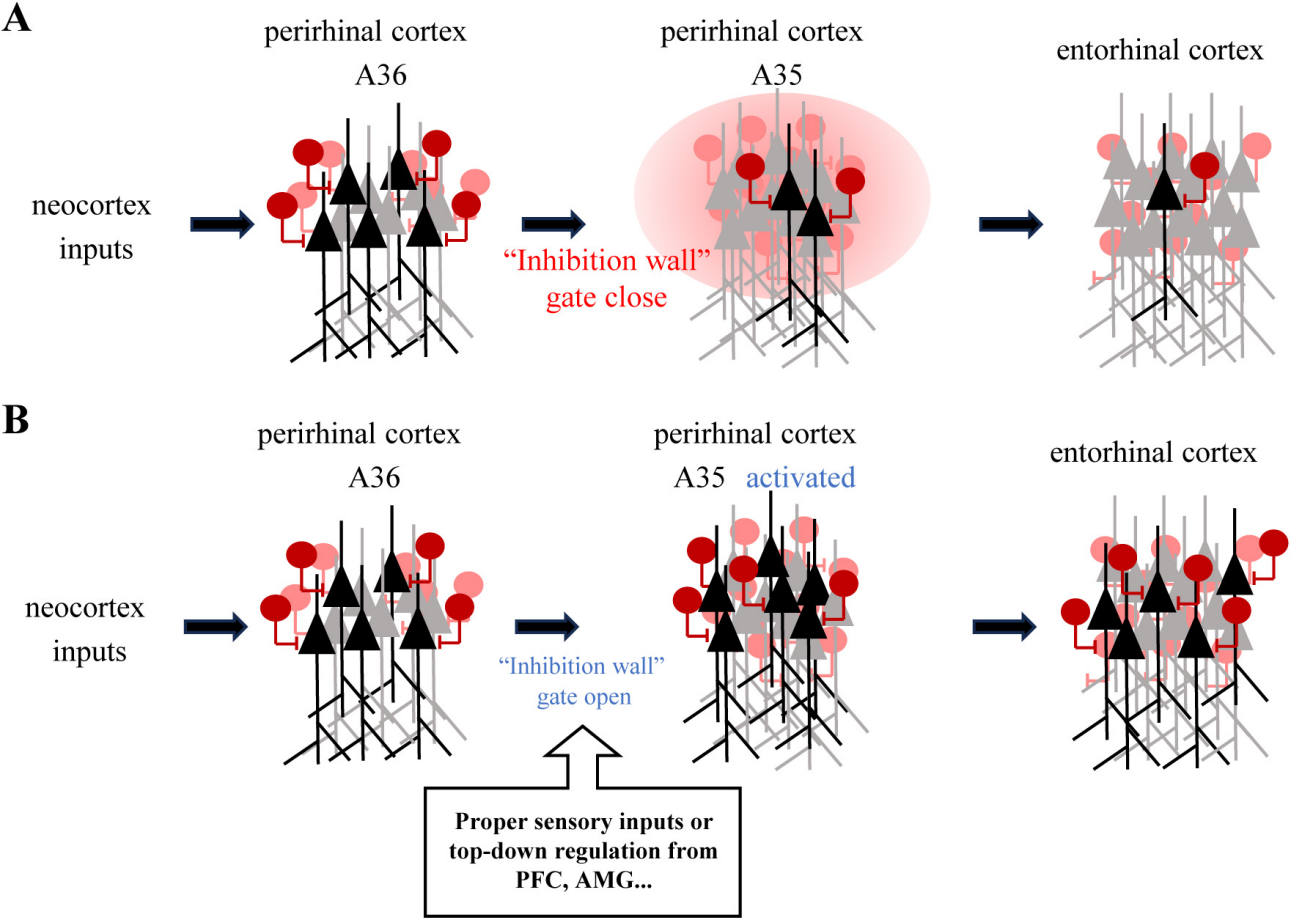
The robust local inhibitory environment within the perirhinal cortex and its function as an information filter. **(A)** The neocortex stimulation activated a small group of neurons in EC, which implied that the “inhibition wall” within the PER. The inhibition wall compromised GABAergic interneurons circuitry which predominantly switched by the CRs (calretinin cells). **(B)** Approximate inputs could open the inhibitory gate and activate PER A35, which would permit selected information to be transmitted to the EC [adapted from Kajiwara and Tominaga (2021)].

Under a regular condition without a trigger (such as the suppression of GABAergic neurons or activating A35 region), the PER functions as an inhibitory barrier that can block excitatory sensory inputs from neocortex to the EC. However, when local inhibitory circuits in the PER were suppressed or high-frequency stimulation was made to the PER, function of this inhibitory barrier could be attenuated and thus restore sensory information transfer from neocortex to the EC (Kajiwara and Tominaga, 2021). Anatomically, the PER can be subdivided into areas A35 and A36, neocortex inputs transmit first into A36, then to A35 and EC. Using voltage-sensitive dye imaging in rodent brain slices, Kajiwara demonstrated that pharmacological blockade of inhibition in PER with Gabazine induced sustained, long-lasting depolarization in previously unresponsive EC and PER A35 regions. In the absence of drugs, alternating 40 Hz high-frequency stimulation (mimicking gamma oscillations) was applied to superficial A36 and deep A35 layers of PER. The results revealed that only deep A35 stimulation activated EC initially, while superficial A36 input failed to do so. However, after repeated alternating stimulation, superficial A36 inputs gradually gained the capacity to drive EC responses. This finding suggests that information transfer along the PER-EC pathway is dynamically regulated by the excitation of PER A35, and gamma-frequency stimulation may progressively enhance the efficacy of superficial inputs by modulating local inhibitory circuits. And the deep A35 layer of PER serves as a critical node for disinhibiting the “wall” and initiating PER-EC communication (Figure 1). Notably, this gating-state alteration exhibited persistence: even after cessation of A35 stimulation, the ability of A36 to activate EC was maintained for over one hour, implying that the underlying mechanism may involve network-level long-term plasticity.

The oscillation we recorded from LFP signals actually illustrate the synchronized activity of a large number of neurons in time and space (Harris Bozer et al., 2017). When groups of neurons in different brain regions need to work together to complete a cognitive task, they achieve “phase synchronization” by adjusting the temporal rhythm of the discharge. This synchronization can effectively reduce the noise of information transmission and improve communication efficiency. Therefore, the observed power enhancement or phase synchronization of LFP oscillations of specific frequencies is interpreted as direct electrophysiological evidence of enhanced functional connectivity and improved coordination between relevant brain regions. The intrinsic low-frequency membrane resonance (1∼2.5 Hz) of PER neurons suggests a delta-band filtering mechanism that aligns computationally with the region’s role in integrating multimodal information over extended time windows for contextual memory formation (Binini et al., 2021). This resonance may serve as a temporal selection mechanism at the single-neuron level, prioritizing the processing of delta-synchronized, potentially behaviorally significant inputs. Although primarily observed *in vitro* and requiring *in vivo* validation, this mechanism potentially complements the PER’s robust inhibitory microcircuitry, where inhibition enables spatial sparsification, delta resonance may provide temporal selectivity. A key question is whether PER employs cross-frequency coupling mechanisms analogous to the hippocampus’s well-established theta-gamma coupling, which is crucial for information packaging and transmission (Torta et al., 2009). Elucidating how potential delta-based interactions in the PER facilitate preliminary information bundling and coordinate with hippocampal theta-gamma dynamics is essential for a complete understanding of the neural mechanisms underlying associative learning.

In summary, convergent evidence from cellular composition, circuit plasticity, and oscillatory dynamics demonstrates that the PER possesses a robust inhibition, mediated predominantly by CR neurons. At the cellular level, the high density of CR neurons in PER facilitates disinhibition of pyramidal cells by suppressing SOM-expressing interneurons, thereby enabling selective gating of information flow. At the circuit level, patterned high-frequency stimulation (e.g., 40 Hz gamma) can induce lasting gate-opening plasticity in deep PER layers, permitting otherwise inhibited signals to propagate to the EC, likely through long-term potentiation mechanisms. Furthermore, the intrinsic membrane resonance of PER neurons in the delta frequency provides a temporal filtering mechanism for integrating multimodal inputs over extended windows. Collectively, these features support a model in which PER serves not as a passive relay but as a dynamic hub for information filtering and selecting. Special regulation of information pass or not from the PER makes the foundation of association formation, and it’s governed by finely-tuned inhibitory microcircuits within the PER.

### 2.2 Association formation in the PER

Traditionally, the PER was regarded as a higher-order visual processing area mediating object recognition memory, with its function often simplified to this core role. However, recent breakthroughs have challenged this perspective, revealing that the functional scope of the PER extends far beyond that of a traditional unimodal visual integration cortex. Anatomically, as a pivotal hub within the medial temporal lobe memory system, the PER converges highly processed information from virtually all sensory modalities, making it an ideal site for the integration of multiple features. Consequently, the functional profile of the PER surpasses object recognition, establishing it as a key node for associative learning (Keene et al., 2016; Naya, 2016; Fiorilli et al., 2021; Burwell, 2024). This section will systematically elaborate on the central role of the PER in integrating diverse information dimensions to form complex associations, following a logical progression from the integration of specific perceptual features to the binding of objective relationships, and ultimately ascending to the representation of abstract cognitive concepts.

#### 2.2.1 Encoding of complex features

The most fundamental and core associative function of the perirhinal cortex lies in integrating discrete visual features to construct coherent, holistic representations of complex objects and enabling fine discrimination between highly similar objects. Neurophysiological studies reveal that PER neurons do not merely respond to discrete elements, such as edges or textures, but preferentially encode global attributes of objects, such as their overall contour and structure, thereby binding multiple features into unified and distinct object representations (Lim and Lee, 2024). This holistic encoding mechanism forms the neural basis for PER’s role in discriminating between highly similar objects.

**FIGURE 2:**
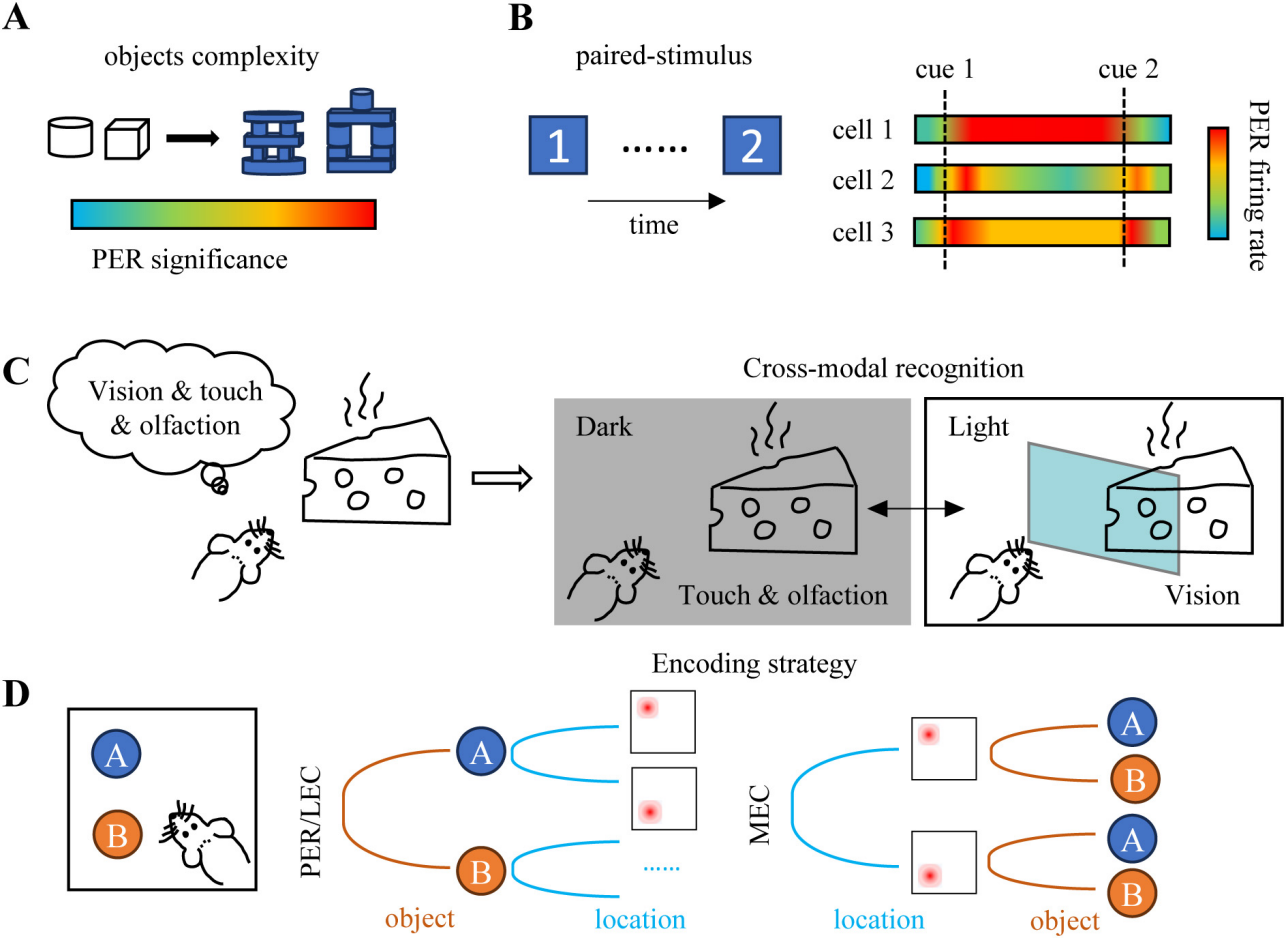
Information integration within the perirhinal cortex. **(A)** PER is more necessary in object recognition tasks for complex objects, less for simple ones [adapted from Norman and Eacott (2004), Bartko et al. (2007)]. **(B)** During the visual paired-stimulus task, neurons in PER encode the learned pairings. Sample cell 1 is a cue-holding neuron, which consists of selective firing during the delay time until presentation of the other stimulus; sample cell 2 is a pair-recall neuron, which represents the to-be-retrieved target (cue2) after sampling of cue 1. These two types of neurons are contained in A36. Sample cell 3 is a unitized neuron within A35, whose response amplitudes are the same between the two stimuli in a pair [adapted from Naya (2016)]. **(C)** The PER was important in multi-sensory unitization and cross-modal recognition tasks [adapted from Albasser et al. (2011)]. **(D)** The PER, LEC and MEC neurons respond to both object and spatial information, but the PER/LEC’s encoding strategy was different from that of the MEC. The PER/LEC first distinguished the object, and the MEC was on the contrary [adapted from Keene et al. (2016)].

Furthermore, the functional importance of PER in object recognition becomes more critical as the complexity or similarity of the objects increases. When discriminating between highly similar object pairs, the activity patterns of PER neurons sensitively reflect subtle feature differences, effectively preventing memory confusion (Figure 2A). Lesion studies consistently demonstrate that PER damage has a more pronounced impact on the discrimination of high-similarity object pairs. For instance, in experiments by Norman, PER lesions impaired the discrimination of block-constructed high-similarity object pairs significantly more than that of low-similarity everyday objects (Norman and Eacott, 2004). Similarly, Bartko et al. (2007) found that PER lesions caused significant behavioral deficits only when the features were sufficiently complex or overlapping, a pattern also validated in olfactory tasks (Feinberg et al., 2012). Human fMRI studies further reveal a functional dissociation between PER and the HPC: PER activation is strongest during conditions of high object similarity, indicating its specific role in fine-grained discrimination, whereas HPC activation is more prominent when objects are categorically different, suggesting its focus on distinguishing between distinct categories (Chen et al., 2019). Overall, the PER is a key region for the transition from local feature processing to holistic object perception, providing the fundamental representational units for higher-order associative learning.

#### 2.2.2 Formation of multimodal and paired-stimulus associations

The associative function of the perirhinal cortex extends beyond the visual modality, establishing it as a critical hub for integrating information across diverse sensory channels (e.g., visual, auditory, tactile) and supporting the formation of paired associations, thereby playing a central role in constructing the foundation of episodic memory. Research indicates that when stimuli from different modalities are repeatedly paired in time, neurons within the PER develop selective responses to this specific associative relationship (Messinger et al., 2001). This learning mechanism applies not only to cross-modal associations but also to associations between different stimuli within the same modality.

At the mechanistic level, the intrinsic functional organization of the PER provides a sophisticated neural substrate for associative encoding. Recordings from PER neurons in macaques performing visual paired-associate tasks revealed that area A36 contains two main types of functionally specialized neuron (Naya et al., 2003; Suzuki and Naya, 2014; Naya, 2016): (1) cue-holding neurons, which maintain selective activity for the cue stimulus throughout the delay period, and (2) pair-recall neurons, which activate approximately 200 ms after the cue1 onset and are thought to encode the to-be-recalled paired stimulus (cue2). In contrast, area A35 contains “unitized neurons” that respond similarly to both stimuli of a paired associate, suggesting the encoding of the stimulus pair as a unified, indivisible representation (Fujimichi et al., 2010; Figure 2B). This functional dissociation indicates that the PER can both discriminate between individual elements within a paired association and represent the integrity of the associative whole.

The role of PER in multimodal integration is further corroborated by studies in rodents. For instance, using complex objects with visual, tactile, and olfactory cues in recognition tasks, scientists found that PER lesions did not cause a global failure in processing all sensory cues but specifically impaired the animal’s ability to effectively utilize and coordinate multimodal information. Rats with PER lesions were impaired in recognition under conditions relying on visual cues but performed normally in complete darkness relying solely on olfactory cues (Albasser et al., 2011). This suggests that the PER’s function lies in integrating and regulating the information flow between different sensory modalities, rather than merely serving as a conduit for any single modality (Figure 2C). In human fMRI study, it was found that the activation of PER was significantly increased when the information in the visual and tactile modes was consistent. It shows that PER is especially important in tasks that require the integration of multimodal information (Holdstock et al., 2009).

Further evidence highlights the experience-dependent plasticity of PER in multimodal integration. Studies show that cross-modal recognition without pre-exposure requires the collaborative effort of the PER and the posterior parietal cortex; however, if animals are pre-exposed to the multimodal information, subsequent cross-modal recognition primarily relies on the PER without significant parietal cortex involvement (Jacklin et al., 2016). Lesion studies confirm the critical role of the PER in fear memory learning that depends on multimodal sensory input (Bartley and Furtak, 2021). *In vivo* electrophysiological recordings demonstrate that the PER contains distinct neuronal populations selectively encoding information from different modalities (Lim and Lee, 2024). Notably, even when identical stimuli are presented, the firing patterns of these neurons differ between unimodal and multimodal task contexts, allowing the discrimination of the current sensory-modality context the animal is experiencing.

Taken together, the PER plays a central role in binding information from different sensory sources into meaningful object-object or object-attribute associations, through its functionally specialized subregions and regulatory role in cross-modal tasks.

#### 2.2.3 Contextual binding in space and time for episodic memory

Traditionally, it has been posited that object information and spatial information are processed separately via PER/LEC pathway and POR/MEC pathway, respectively. However, this paradigm of functional segregation is being challenged. A pivotal perspective is that the core function of PER extends beyond mere object information relay, positioning it as a high-level association center whose integrative capacity is fundamental for forming episodic memories with specific spatiotemporal contexts. Evidence supporting this view stems from anatomical studies, which have revealed dense reciprocal connections between PER and POR (Burwell et al., 1995; Burwell and Amaral, 1998b; Furtak et al., 2007). These anatomical linkages suggest that, prior to the information flow into HPC, PER and POR may cooperate in the binding and integration of cross-modal information. Early lesion studies demonstrated that PER is essential for tasks with spatial memory which involve ambiguous cues or combining object recognition. PER damage also leads to decreased stability of hippocampal place fields, disrupted theta-phase locking, and impaired correlations between running speed and neuronal firing rates, indicating a critical role for PER in maintaining spatial memory stability, encoding precision, and path integration (Aggleton et al., 2004). Consequently, PER not only processes complex object features but also, through its interactions with adjacent regions, integrates spatial, temporal, multisensory information, thereby providing pre-associated informational groundwork for the subsequent formation of coherent episodic memories by HPC.

More evidence supports PER’s involvement in spatiotemporal integration. Lesion study indicated that PER involved in object-context or object-location-context memory (Barker and Warburton, 2020). Furthermore, a key finding is that the encoding properties of perirhinal cortical neurons demonstrate that this region is capable of not only representing detailed object features but also forming coarse representations of the macro-spatial context (Bos et al., 2017; Burwell, 2024). Specific evidence reveals that in rats, neurons in PER, LEC, and MEC all respond to both object and spatial information, but their encoding strategies differ: PER/LEC neurons show high selectivity for different objects in the same location, while maintaining similar responses to the same object across locations; in contrast, MEC coding is more spatially oriented (Keene et al., 2016; Figure 2D). Moreover, PER neurons can distinguish macro- spatial segments (e.g., left vs. right maze arms) through sustained firing. Although not necessary for pure spatial navigation, PER is crucial for scene-guided object recognition memory. In non- human primates, PER neurons can integrate object- location information and exhibit response similarity to different objects that have been presented in the same location, this similarity emerges earlier in PER than in other regions, suggesting that PER may be the earliest site for extracting object- place associations (Chen and Naya, 2020; Yang and Naya, 2023).

Beyond spatial integration, PER contributes to organizing objects, actions, and other elements along the temporal axis into coherent events. In rat eyeblink conditioning, sustained firing in PER during timed intervals may mediate delayed associative learning (Suter et al., 2013). In monkey studies, PER neurons not only encode stimulus- reward associations but also incorporate temporal- context information to form broader linkages (Eradath et al., 2015). Furthermore, PER neurons modulate their firing rates to distinguish the same stimulus presented in different temporal sequences (Naya, 2016; Naya et al., 2017). These findings collectively highlight PER’s role in temporal coding and event structure.

PER also plays a regulatory role in memory consolidation and extinction. Research indicates that protein synthesis within PER is critical for the consolidation and extinction of cross- modal associative memories in neutral contexts (Balderas et al., 2013). During early memory phases, paired memory engrams co-exist in both PER and the basolateral amygdala. And memories are subsequently selectively consolidated depending on contextual safety: in safe contexts, PER engrams are strengthened while BLA engrams are suppressed; with the opposite pattern occurring in dangerous contexts (Qureshi et al., 2023).

Given these observations, PER elevates simple object associations to the level of complex event representations by binding “what,” “where,” and “when” information, thereby serving as a key hub for constructing richly detailed episodic memories or involving in distinct phases of memory process such as consolidation or extinction. Its function extends beyond that of a mere sensory gatekeeper, positioning PER as a central nexus for multimodal associative formation that supports the encoding and organization of high- order episodic memory.

#### 2.2.4 Representing of abstract concepts

Building upon its role in integrating objective sensory features, the PER further represents a suite of abstract concepts based on previous experience, including judgments of stimulus familiarity or novelty, reward-based motivational value, and experience-based predictions about future outcomes (Ohyama et al., 2012; Suzuki and Naya, 2014; Lee et al., 2024). These functions underscore the role of PER as a hub for high-level association formation, bridging concrete sensory inputs with internal states such as motivation, emotion, and expectation.

The PER is critical for familiarity-based recognition memory, but its core function is not merely the detection of “novelty” *per se*; it rather involves associating the novelty signal with specific sensory objects. This view is supported by converging evidence. For instance, early studies demonstrated that PER neurons respond more strongly to novel than to familiar visual stimuli, forming a putative neural substrate for familiarity judgment (Xiang and Brown, 1998; Wan et al., 1999); and the HPC neurons respond more strongly to novel arrangement. Based on such findings, Aggleton and Brown proposed the “double dissociation model” of recognition memory, separating familiarity judgment (dependent on PER) from recollection-based associative matching (dependent on HPC) (Brown and Aggleton, 2001). Subsequent work in non-human primates also showed that PER neuronal responses become smaller as stimulus repeats, creating a “familiarity signal” (Suzuki and Naya, 2014). However, more nuanced behavioral experiments revealed that while PER lesions impair discrimination when novel and familiar objects are presented together, they do not reduce initial exploration of a novel object alone or the reduced responses upon repeated presentation of a single object (Albasser et al., 2011, 2015; Olarte-Sánchez et al., 2015). This collection of evidence indicates that the essential role of PER is to bind the abstract feature of “novel” or “familiar” to a specific perceptual entity, rather than generating a global novelty signal.

On the other hand, the PER is actively involved in assessing the motivational and affective value of stimuli. Its neuronal activity reflects the predicted reward value (both positive and negative) of stimuli and integrates sensory features to form stable value associations. Studies in macaques demonstrated that PER lesions impair the ability to learn visual cue-reward associations and abolish the gradient of performance improvement as reward proximity increases (Liu et al., 2000). Crucially, PER neurons can encode not only single stimulus-reward associations but also flexibly represent reward expectations predicted by combinations of stimuli in complex contexts (Ohyama et al., 2012). This process likely relies on functional connectivity between the PER and the orbitofrontal cortex, as their interaction is necessary for generating accurate expected value estimates (Clark et al., 2013). Electrophysiological recordings in rodents further delineate the dynamic role of PER in value association. For example, in rats performing an object-cued choice task, many PER neurons increased firing following the choice of a specific item, indicating the integration of object information, behavioral choice, and outcome feedback (Ahn and Lee, 2015). In a sophisticated spatial delay-choice task, rats with inactivated PER failed to establish a stable choice strategy, showing decisional hesitation and behavioral inflexibility. This suggests that PER is necessary for stabilizing the association between choices and their outcomes in a dynamic environment, thereby supporting adaptive decision-making (Kreher et al., 2019). Other studies found that PER neuronal activity peaks as animals approach a reward location, and its encoding of “sensory feature-reward associations” strengthens gradually during learning (Fiorilli et al., 2024; McLachlan et al., 2025). Collectively, these findings establish the PER as a critical hub for forming and maintaining associations between stimuli and their motivational significance.

Extending beyond representing current value, neural networks within the PER can generate predictions based on established associations. Their activity patterns can reflect prediction errors (the discrepancy between actual and expected outcomes) and support the abstraction and generalization of behavioral rules, functioning as an internal “predictive model.” Evidence shows that a subset of PER neurons specifically encodes reward prediction errors. For instance, their activity increases significantly following an erroneous choice and can predict behavioral adjustments in subsequent trials (Ahn and Lee, 2015; Fiorilli et al., 2024). During learning, the time at which PER neurons decode reward information gradually shifts earlier, indicating that stable stimulus-reward associations are internalized into the predictive framework of PER, allowing it to generate outcome expectations even before behavior is initiated (Lee et al., 2024). At a more advanced level, the PER contributes to rule abstraction. When animals learn a new task that shares the underlying rule but differs in sensory details from a previously mastered task, the neural representations for the old and new tasks in PER gradually converge from initial separation, eventually mapping onto similar neural subspaces. This demonstrates the PER’s capacity to encode abstract task structures that transcend specific sensory details (Lee et al., 2024). Furthermore, in primate object-location association tasks, two types of integrative neurons were identified in PER: one type exhibits location selectivity before stimulus presentation, subsequently adding object information to this spatial framework; the other type specifically binds an object to its location only after the stimulus appears (Chen and Naya, 2020). The pre-configuring activity of the former type of neuron exemplifies the PER’s ability to utilize prior information (e.g., spatial context) to form predictive representations.

Based on the above evidence, the PER is deeply involved in processing abstract concepts by representing familiarity, motivational value, and generating contextual predictions. It links concrete sensory inputs, internal states (such as motivation and expectation), and behavioral context, providing a critical computational substrate for higher-order cognition and behavior.

#### 2.2.5 Cognitive control over associative formation

The associative formation in PER is not a fully automatic or rigid process; it is rather finely regulated by top- down cognitive control, such as attention, ultimately giving rise to “task- related unit” representations. Specifically, depending on behavioral goals, attentional resources dynamically modulate PER activity, enhancing the selective representation of task- relevant information while suppressing irrelevant inputs, thereby optimizing associative encoding. Supporting evidence comes from an attentional interference paradigm in rats (Trettel et al., 2021): PER lesions led to reduced accuracy and prolonged learning during the acquisition phase; impulsive responses increased, and omission rates were higher under noisy conditions that challenged attention. These outcomes indicate that top-down attention regulation promotes the formation of task-related units in PER, and PER is involved in the correct retrieval of memory, which controls impulsive response and omission rates. PER damage impairs attentional control, which is likely mediated by top- down pathways from the PFC that jointly promote goal- directed behavioral selection and inhibition.

Taken together, PER not only integrates multidimensional features of complex objects to enable fine discrimination but also binds cross- modal, spatiotemporal, and contextual information, thereby constructing representational networks that support abstract concepts. Based on these functions, Fiorilli et al. (2021) proposed that the core function of PER is to integrate multiple features–such as object properties, spatial locations, sensory modalities, and affective valence–into a unified, behaviorally meaningful “task- related units” (Fiorilli et al., 2021). This capability relies on several mechanisms: anatomically, PER receives convergent inputs from multiple high- level sensory cortices, providing a substrate for feature integration; at the circuit level, strong local inhibition and complex excitatory- inhibitory interactions within PER enable selective responses to different feature combinations; and at the network level, dynamic interactions among PER, HPC, amygdala, and PFC allow behaviorally relevant associative networks to be preferentially strengthened or retrieved according to ongoing cognitive demands.

## 3 PER-HPC network circuit and how information flows in it

### 3.1 Anatomical architecture of the parahippocampal-hippocampal network circuit

The perirhinal cortex, comprising Brodmann areas 35 and 36 within the medial temporal lobe, serves as a pivotal component of the parahippocampal region (Kealy and Commins, 2011; Beaudin et al., 2013). It exhibits distinct heterogeneity in both cytoarchitecture and connectivity patterns. While the PER primarily integrates multimodal inputs from temporal association cortices, the neighboring POR is more involved in spatial processing. Within the dual-pathway model of memory systems, the PER constitutes a core node of the ventral “what” stream, responsible for transmitting object recognition information to the hippocampus (Wan et al., 1999). This pathway–via PER-LEC-hippocampus–operates in parallel with the dorsal “where” stream (via POR-MEC-hippocampus). Importantly, the PER and LEC exhibit dense bidirectional connectivity, with topographically organized projections (de Villers-Sidani et al., 2004).

Emerging evidence indicates that PER-HPC communication not only involves indirect pathways through EC but also parallel direct projections (Kosel et al., 1983; Naber et al., 1999; Huang et al., 2021). Anterograde tracing and optogenetic studies have identified monosynaptic connections from PER to hippocampal CA1 and SUB, which may preferentially target inhibitory interneurons rather than pyramidal cells (Li et al., 2019). Moreover, task-dependent routing mechanisms refine the dual-pathway model: novel object information may flow through the indirect pathway (PER-LEC-DG-CA3-CA1), whereas familiarity judgments engage the direct LEC-CA1 route for rapid processing (Albasser et al., 2009; Kinnavane et al., 2016). These findings highlight the dynamic and context-dependent nature of PER-HPC circuitry.

Beyond the traditional “what/where” dichotomy, a refined “detailed/coarse” network model has been proposed. In this framework, the indirect pathways from PER/POR, via EC and DG/CA3, are posited to convey coarser, gist-like information. In contrast, the direct projections from these regions to CA1 are thought to transmit more detailed, high-resolution featural information. This architectural specialization supports the efficient integration of object identity with spatial context.

**FIGURE 3:**
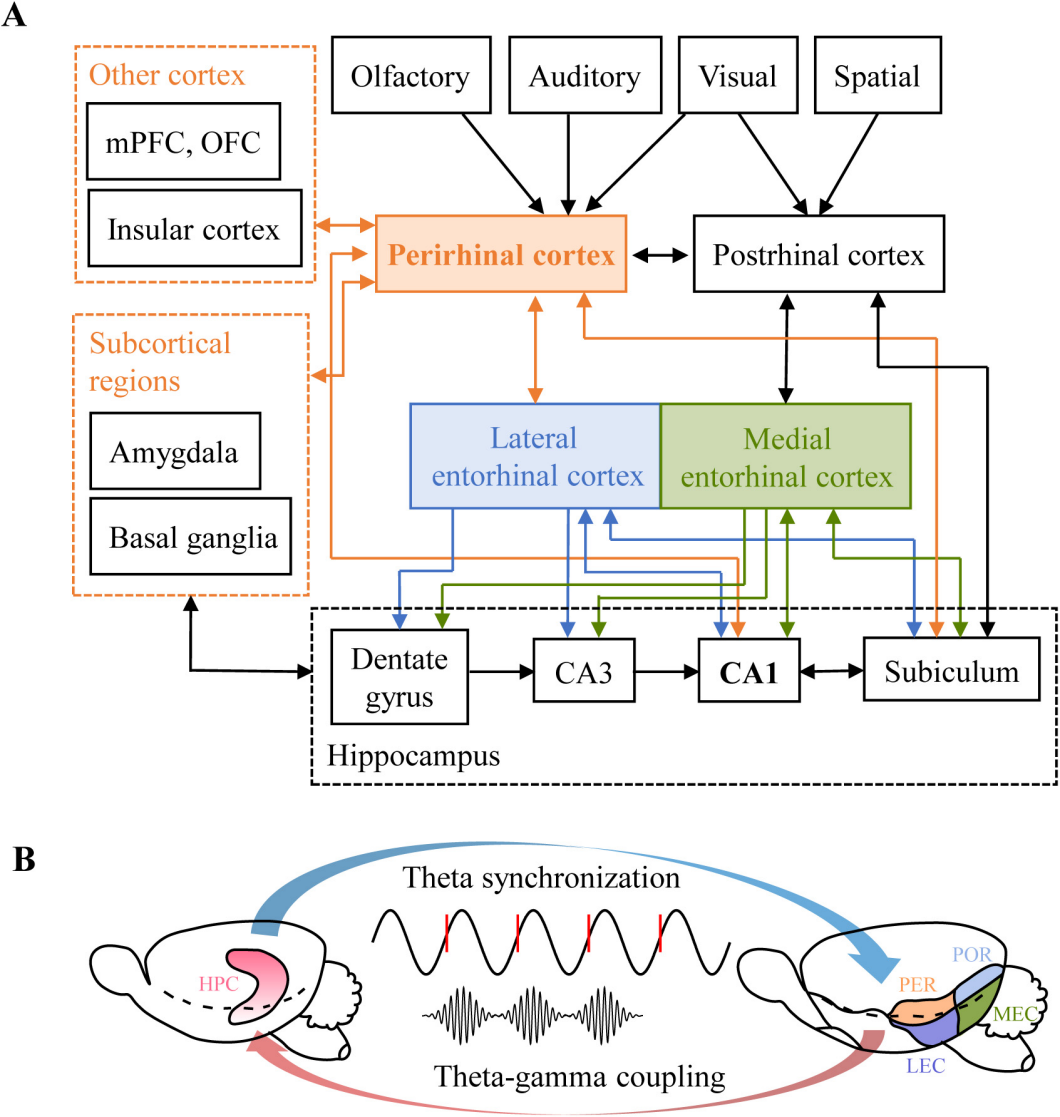
The anatomic connections of the hippocampal-parahippocampal network. **(A)** The schematic overview of the main anatomical connections of the para-hippocampus network, especially the inputs and outputs of the perirhinal cortex. **(B)** The diagram of oscillation synchronization between the HPC and PER. Theta oscillation synchronize from the HPC to that of the PER during learning behavior. PER spikes gradually show phase locking with HPC theta. And the coupling gamma oscillation conveys information efficiently to the HPC [adapted from Bos et al. (2017)].

These extensive connections support PER’s role in integrating object recognition, contextual associations, and memory modulation, rather than merely functioning as a passive relay station. By projecting directly to the EC and hippocampal subfields such as the subiculum (SUB) and CA1, the PER serves as a critical gateway for conveying high-order sensory information to the hippocampus (Figure 3A). The functional integration within the PER-HPC circuit is critical for episodic memory formation (van Strien et al., 2009; Kealy and Commins, 2011). The PER not only consolidates multimodal features (e.g., object, temporal, and contextual information) but also gates information flow via local inhibitory microcircuits. This regulation enables the delivery of information at varying levels of abstraction to downstream hippocampal targets, thereby facilitating the encoding of novel associations.

In summary, converging evidence positions PER not as a passive information relay but as an active associative formation center. Through its intrinsic microcircuits and parallel output pathways, it actively binds multimodal features into representations of differing abstraction levels and directs these representations to distinct hippocampal subregions. This process efficiently drives the final integration of information within the hippocampus, which is crucial for the formation of coherent episodic memories.

### 3.2 Mechanisms of information flow in the PER–HPC circuit

Advances in behavioral, optogenetic, and electrophysiological techniques have progressively elucidated the functional relationship between the PER and HPC. Studies demonstrate that these two regions exhibit bidirectional dependency in association formation, thereby damage to either structure significantly disrupts memory integration (Aggleton et al., 2004; Yanike et al., 2009).

#### 3.2.1 Direct information transfer within the circuit

The PER and HPC engage in close bidirectional communication during memory encoding and retrieval. Critical evidence for their functional interdependence comes from lesion studies. PER damage impairs spatial coding in the HPC, whereas HPC damage compromises object recognition functions in the PER (Allen et al., 2020). This reciprocal disruption underscores that the perirhinal-hippocampal circuit is essential for bidirectional information transfer, with each structure supporting the functional integrity of the other.

Functioning as a primary gateway for high- order sensory information entering the hippocampus, PER damage undermines the HPC’s ability to receive detailed sensory input. For instance, in rodent object- location association tasks, PER lesion not only increases the drift of place fields in HPC place cells after a delay but also disrupts the positive correlation between HPC firing rate and running speed, as well as the phase- locking of HPC activity to theta oscillations, thereby compromising spatial memory stability and path integration (Aggleton et al., 2004).

Primate research further clarifies the directionality and timing of information flow. During object- location memory retrieval in monkeys, location- specific neural activity emerges first in the PER, followed by the hippocampus and parahippocampal cortex, suggesting that memory recall is initially triggered in the PER before being relayed to the HPC (Yanike et al., 2009; Yang and Naya, 2023). Human fMRI studies corroborate this functional coupling: the functional connectivity between left PER and HPC strengthens over learning, and its strength negatively correlates with forgetting rates, implying that the intensity of PER-HPC interaction is closely linked to memory consolidation (Vilberg and Davachi, 2013).

Further refinement of these pathways comes from studies on their intermediate nodes and functional segregation. Lesions of LEC, a primary relay from PER to HPC, impair associative memory while sparing non-associative memory, suggesting its role in conveying coarse, gist-like associative information (Wilson et al., 2013). Conversely, PER lesions impair the ability to distinguish between highly similar objects, indicating that the direct projections from PER to CA1 are critical for transmitting fine-grained, detailed object information. Conversely, HPC damage also impairs associative encoding in the PER. In animals with HPC lesions, PER neurons retain responsiveness to individual objects but lose the ability to encode object- space or object- time associations (Allen et al., 2020). Moreover, “time cells” in the HPC, which exhibit monotonic firing rate changes during delay periods, may provide critical temporal information for the PER to integrate temporal context (Naya et al., 2017). Together, these findings indicate that the PER and HPC engage in bidirectional, interdependent information flow during memory processing. the PER contributes detailed feature information and early memory reactivation, while the HPC provides spatial and temporal context. Their cooperative interaction, mediated by both direct and indirect pathways with distinct informational roles, is fundamental for the encoding and retrieval of coherent associative memories.

#### 3.2.2 Oscillatory synchronization between PER and HPC

Neural oscillatory synchronization is a key mechanism mediating long- range communication between brain regions. During memory processing, the PER and HPC coordinate their activity through coupled theta and gamma rhythms to facilitate information transfer and integration.

Theta oscillations provide a temporal window for PER–HPC information exchange. During exploration and learning, theta rhythms in HPC synchronize with theta activity in the PER. This synchronization is thought to coordinate the transmission of spatial- contextual signals from the HPC to the PER, thereby tagging the locations of object occurrences (Bos et al., 2017). During visual discrimination tasks, PER neuronal spiking becomes phase- locked to HPC theta oscillations, suggesting that the HPC may rhythmically regulate information flow in the PER (Figure 3B).

Furthermore, HPC theta acts as a global pacemaker, propagating through the LEC to the neocortex and guiding the enhancement of gamma synchrony in cortical areas, thereby promoting the integration of local information (Luo et al., 2022). Gamma oscillations are hypothesized to “package” object- feature information encoded in the PER and convey it efficiently to the HPC within theta cycles (Lisman and Jensen, 2013). This theta- gamma coupling is considered crucial for memory encoding.

During memory consolidation, sharp- wave ripples (SWRs) play a particularly important role. SWRs, predominantly occurring during rest or sleep, involve the “replay” of recently encoded memory sequences in the HPC. Evidence indicates that during synchronized cortical up-states, hippocampal SWR activity is associated with enhanced global cortical excitability (Headley et al., 2017). Specifically, compared to up-states devoid concurrent hippocampal events, SWR-coincident up-states exhibit a significant increase in neuronal firing rates and local gamma oscillation power across multiple cortical regions, including the PFC, sensory cortex, PER and EC. Notably, this enhancement in gamma power displays a distinct spatiotemporal gradient, with activity propagating sequentially from the PFC-sensory cortices-PER-EC, indicating that these high- frequency events may drive the reorganization of information between the forebrain and sensory areas during systems consolidation. Nevertheless, the precise neural circuitry through which SWRs trigger widespread cortical gamma power increases still remains unclear.

#### 3.2.3 Coordinated modulation by other brain regions

The PER-HPC circuit does not operate in isolation, its information processing is modulated by multiple extrinsic inputs, most prominently the PFC, amygdala, and neuromodulatory systems.

The PFC plays a central role in regulating this circuit. As a cognitive control hub, the PFC can adjust information flow within the PER-HPC pathway according to behavioral goals. Direct bidirectional anatomical connections exist between the PFC and PER, and these connections are organized according to a specific topography. Specifically, distinct subregions of PFC (such as the medial PFC and the orbitofrontal cortex) exhibit finely tuned and specific projection patterns to different areas of PER. PFC sends dense descending projections to PER, with axons predominantly terminating in its infragranular layers. Concurrently, PER provides ascending feedback projections to PFC (particular the mPFC and anterior cingulate cortex), mainly synapsing in the supragranular layers of PFC (Deacon et al., 1983; Bedwell et al., 2015).

Lesion studies show that contralateral damage to the HPC with medial PFC and PER in rats causes more severe deficits in object-location-context association than ipsilateral damage, indicating that these three regions must cooperate to support associative learning (Warburton and Brown, 2010; Barker and Warburton, 2020). Pharmacological inactivation experiments provide causal evidence that blocking the PFC-PER pathway significantly impairs behavioral flexibility in animals. Specifically, inhibition of this pathway renders animal incapable of updating object selection rules based on changes in spatial location. This finding demonstrates that when environmental rules change, PFC engage in real-time communication with PER to suppress old, habitual response patterns and extract new associative information to guide adaptive behavior flexibly (Hernandez et al., 2017). The PFC-PER pathway also critical for sequence memory. By modulating perirhinal activity, PFC aids the brain in encoding and recalling the temporal order of events, a core feature of episodic memory. Research indicates that PFC projections to PER can regulate how PER neurons encode temporal intervals and order, thereby directly supporting the recall of event sequences (Jayachandran et al., 2019). Furthermore, the mPFC regulates context-guided memory reconsolidation in the PER by utilizing serotonin 2a receptor signaling to select the most context-appropriate memory trace for updating (Morici et al., 2018, Morici et al., 2022a).

The PFC and HPC are interconnected through both direct and indirect neural pathways, forming a fundamental circuit for advanced cognition. As a key top-down regulator, the PFC exerts modulatory control over hippocampal processing. Anatomically, a prominent direct pathway exists from mPFC to HPC, with fibers projecting via the fornix and terminating in the vHPC and SUB. Conversely, HPC sends return projections back to PFC, primarily targeting the medial and orbital regions, thereby establishing a reciprocal loop. Additionally, influential indirect pathways are mediated through the thalamic reuniens nucleus and EC, which provide essential conduits for PFC signals to influence HPC circuits (Schlichting and Preston, 2016; Eichenbaum, 2017). This indicates connectivity allows PFC to selectively gate and coordinate information flow into and out of HPC.

PFC-HPC interactions are functionally critical for memory integration and the cognitive control of recall. A core function of this circuit is to support the assimilation of new experiences into existing knowledge networks. PFC guides this process by biasing HPC activity to extract shared elements across related events, thereby enabling the formation of generalized schemes (Schlichting and Preston, 2016). Furthermore, this pathway is indispensable for strategic organization and retrieval of episodic memories. The PFC, particularly it medial sector, engages in dynamic, task-dependent synchronization with hippocampal theta oscillations (Morici et al., 2022b). This oscillatory coupling is causally linked to the successful recall of temporal sequences and contextual details, facilitating the reconstructions of coherent episodes from memory (Hwang et al., 2018). During memory-guided decision-making, the PFC also modulates hippocampal representations to suppress irrelevant information and focus on goal-relevant associations. Pharmacological disconnection of the PFC-HPC circuit severely disrupts this ability, leading to contingencies change (Chao et al., 2020).

In summary, PFC acts as a high-level control hub that regulates the PER-HPC circuits by coordinately modulating perceptual feature association and spatiotemporal memory integration through parallel PFC-PER and PFC-HPC pathways, thereby ensuring the adaptability, accuracy, and strategic nature of associative memory formation, retrieval, and updating.

In addition, the amygdala, via its basolateral nucleus, provides emotional inputs to both PER and hippocampal CA1, modulating the encoding of emotional memories. Dopaminergic projections from the ventral tegmental area to CA1 regulate synaptic plasticity, while the basal forebrain cholinergic system (e.g., the medial septum) innervates the HPC and modulates theta rhythms and learning. Together, these neuromodulatory systems shape the activity patterns of the PER-HPC circuit to adapt to varying behavioral and cognitive demands.

## 4 The hippocampal CA1 region: the final integrator and hub for episodic memory

### 4.1 Convergence and integration of information in CA1

The hippocampal CA1 region serves as a critical hub for information integration, converging and processing inputs from multiple brain areas, and is a core node for the formation of coherent episodic memories and spatial representations (Chen et al., 2024). Its pyramidal neurons primarily receive two major streams of input: projections from CA3 via Schaffer collaterals, terminating in the stratum radiatum (SR) and conveying pre-processed associative learning, spatial context, and internal state information from the intra-hippocampal circuit; and projections from the EC, terminating in the stratum lacunosum-moleculare (SLM), delivering immediate, novel sensory (both spatial and non-spatial) information from the external world. CA1 neurons compare and integrate these two streams to generate coherent behavioral commands, which are fed back to the EC and other widespread regions. This process is considered fundamental for memory encoding, updating, and retrieval (Carr and Frank, 2012).

**FIGURE 4:**
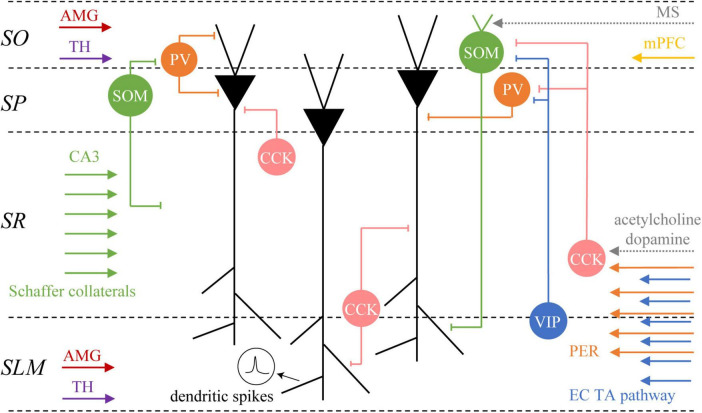
The integration of information in hippocampal CA1 and the microcircuits of interneurons. The schematic diagram of CA1 information integration and microcircuits. Interneurons included in microcircuits contain PV cells (orange), SOM cells (green, included OLM neurons), CCK cells (pink, including basket cells, dendritic-targeting cells and interneuron selective interneurons), and VIP cells (blue). Colorful arrow lines indicate the inputs from different origins. AMG, amygdala; TH, thalamus; MS, medial septum; TA, temporo-ammonic pathway.

Information from the main pathway of the Schaffer Collaterals to CA1 undergoes pre-processing by the DG and CA3. The DG, as the primary input region of HPC, employs pattern separation, utilizing its vast population of granule cells to perform sparse coding, thereby differentiating incoming memory information. In contrast, neurons within CA3 possess extensive recurrent collateral connections, forming the hippocampus’s largest auto-associative network responsible for pattern completion. This network reconstructs partial inputs into complete memory schemas, serving as a fundamental basis for associative memory. In process-based model, the coarse network transmits “gist” information preprocessed by the PER and EC, while the DG and CA3 establish the corresponding associative memory framework (Burke et al., 2018). CA1 acts as an integration node, receiving both this associative memory framework via the SC and detailed information via direct inputs. By dynamically adjusting and balancing these incoming streams, CA1 constructs a unified representation encompassing information at different levels. Beyond this hippocampal-parahippocampal network, research reveals a more complex input network to CA1, which also receives modulatory inputs from the thalamus, amygdala, PFC, and medial septum (Figure 4). For instance, valence signals from the amygdala can enhance the encoding strength of emotionally salient events in CA1, promoting memory consolidation; and the PFC provides top-down cognitive control, involved in goal-directed information selection and working memory maintenance.

#### 4.1.1 Dendritic computation and initial filtering

The dendrites of CA1 pyramidal neurons are the first site for complex computations. Each neuron possesses numerous dendritic spines, receiving approximately 25,000–30,000 excitatory synapses (Andersen, 1990). Local activation of these synapses, distributed across different dendritic compartments, can generate dendritic spikes (e.g., sodium, calcium, or NMDA spikes). This represents a key mechanism for non-linear summation and local gain amplification of inputs (Katz et al., 2009). For example, inputs from the LEC often elicit dendritic spikes but are usually insufficient to independently drive somatic action potentials. Specific interneurons, such as VIP and CCK interneurons, regulate this dendritic excitability through precise inhibitory microcircuits, thereby filtering signals with sufficient spatiotemporal coherence for propagation to the soma and participation in final integration (Bilash et al., 2022).

#### 4.1.2 Plasticity mechanisms and dynamic modulation at the circuit level

At the circuit level, a core mechanism for integrating different inputs in CA1 is spike-timing-dependent plasticity (STDP). When CA3 inputs in the SR and EC inputs in the SLM co-activate the same CA1 neuron within a proximate time window, they can efficiently induce long-term potentiation, specifically strengthening these associated synaptic connections (Piskorowski and Chevaleyre, 2011). This process is dynamically modulated by various neuromodulators: acetylcholine released during exploration of novel environments can promote specific gamma oscillations, creating a temporal window favorable for STDP and preferentially enhancing the flow of novel information from the EC (Fuchsberger and Paulsen, 2022); whereas dopamine bursts following reward acquisition can, via its tonic and phasic firing modes, inhibit or enhance CA3 inputs, respectively, thereby selectively consolidating reward-related memory engrams (Rosen et al., 2015; Figure 4).

Memory consolidation relies not only on instantaneous synaptic modifications but also on system-level reorganization across brain regions and time scales. In this process, the hippocampal CA1 region serves as a critical “memory index” (Tanaka and McHugh, 2018). According to the retrospective projection theory (Renart et al., 1999; Rolls, 2015; Rolls and Kesner, 2016; Rolls et al., 2024), during the early stages of memory formation, the hippocampus (particularly CA1) binds sensory information from disparate cortical modules and rapidly stores a concise “index code.” Over time, during offline states such as sleep (especially slow-wave sleep), the hippocampus repeatedly replays this memory index via synchronized oscillatory activity. This replay gradually guides and strengthens the connections among specific neuronal ensembles distributed in the neocortex, ultimately systematically consolidating the memory engrams within the long-term storage networks of the neocortex. Thus, the hippocampal CA1 region acts not as the final repository of memory but as a navigation center that guides the transformation of long-term memories, a function highly dependent on its retrospective projections to the cortex.

#### 4.1.3 Multi-dimensional representation and dynamic coding at the functional level

The integrative function of the CA1 region is prominently demonstrated in its multidimensional dynamic representations. In spatial navigation, ensembles of CA1 place cells form cognitive maps of environments. Studies show that the proportion of place cells with stable place fields (“persistent cells”) increases with learning, indicating continuous consolidation of spatial representations with experience (Vaidya et al., 2025). Furthermore, CA1 neurons can flexibly integrate spatial information with goal value (Qian et al., 2025). In reward-containing environments, some neurons remap their place fields to be centered around the reward location. Moreover, in novel environments, the overall population representation initially favors goal-related information, rebalancing as the environment becomes familiar. This dynamic weighting of different information sources may involve mechanisms like behavioral timescale synaptic plasticity (BTSP), which allows the association of significant events with specific locations over extended, second-long time windows via sustained dendritic plateau potentials.

CA1 function extends beyond physical space into the domain of social cognition (Watarai et al., 2025). The ventral CA1 harbors “social attribute cells” and “social identity cells” that encode features like sex and strain of conspecifics, and integrated representations of specific individuals, respectively. These representations are organized via precise firing timing within the theta rhythm. Finally, CA1 continuously integrates allocentric (world-centered) and idiothetic (self-motion) information, showing a preference for egocentric coding (Yang and Naya, 2023; Zhang et al., 2024). For instance, during object-place association tasks, the allocentric object-place association conveyed from the PER is integrated with the default spatial background map within the hippocampus to form an egocentric mental image suitable for path planning. This ability to flexibly switch and integrate dominant information sources according to behavioral demands is a hallmark of CA1 as a high-level integration center.

### 4.2 Fine-tuned regulation of cell assemblies in CA1 by interneurons

The precision of information integration in the hippocampal CA1 region relies heavily on the sophisticated regulation by local inhibitory microcircuits. Evidence indicated that some of the interneurons specially receive inputs and regulation from other regions, such as the PER, unlike the LEC, prefers to target on interneurons within SR/SLM layers (Li et al., 2019). Inhibitory interneurons in this region exhibit high heterogeneity. Through specific connectivity patterns, synaptic targeting, and firing timings, they precisely govern the integration of excitatory inputs, spike timing, and synchronization of PCs, thereby collectively shaping the cell assemblies that encode memory representations (Pelkey et al., 2017; Jeong and Singer, 2022). This regulation is highly coordinated in the temporal domain, with distinct interneuron types firing at specific phases of brain oscillations (e.g., theta, ripples) across different brain states, forming a dynamic inhibitory matrix. Beyond the primary inputs from CA3 and EC, the CA1 region receives diverse inputs from the amygdala, medial septum, thalamus, and PER, which interact with specific interneuron microcircuits, further enriching the computational capabilities of CA1 (Li et al., 2019; Topolnik and Tamboli, 2022).

#### 4.2.1 Parvalbumin (PV)-expressing interneurons

Parvalbumin-positive interneurons primarily target the somas and proximal dendrites of CA1 pyramidal cells. Known for their fast-spiking properties, they provide powerful and rapid inhibitory input to PCs. Among them, basket cells contribute to generating and regulating network rhythms (e.g., gamma oscillations) through periodic, population-wide inhibition-release cycles (Topolnik and Tamboli, 2022). And PV basket cells are a critical component of “feed-forward inhibition.” Axo-axonic cells (chandelier cells) specifically target the axon initial segment of PCs, acting as the ultimate “gatekeeper” for action potential generation. They precisely control the output timing of PCs and play a key role in coordinating large-scale network synchronization and behavior-dependent network reorganization (Dudok et al., 2021). For example, optogenetic inhibition of chandelier cells could impact the remapping of CA1 place cells. Bistratified cells innervate both apical and basal dendritic regions of PCs. Their extensive axonal arborization allows them to simultaneously modulate the integration of inputs from both CA3 (Schaffer collaterals) and EC (temporo-ammonic pathway).

Collectively, PV cells can accurately coordinate the integration of different input information in time, affecting the synaptic plasticity and rhythm generation of the network. Furthermore, studies indicate that reduced activity of PV interneurons in novel environments dampens overall network inhibition, thereby facilitating the encoding of new memories (Hainmueller et al., 2024). Reduced activity of PV interneurons in different environments could lead to increased PC activity, and then improve the correlation of neuronal representations of different contexts.

#### 4.2.2 Somatostatin (SOM)-expressing interneurons

Somatostatin-positive interneurons constitute another crucial regulatory population in CA1. The oriens-lacunosum moleculare (OLM) cell is the most studied subtype. Its soma resides in the stratum oriens (SO), and its axons project to the SLM, specifically inhibiting the distal apical dendrites of PCs, where EC inputs arrive. OLM cells form a key component of feedback inhibitory loops, receiving excitatory input from CA1 PCs and subsequently regulating the integration of EC inputs with CA3 inputs on PC dendrites (Rolotti et al., 2022; Udakis et al., 2025). In addition to inputs from PCs, OLM neurons receive cholinergic input from the medial septum, which could regulate OLM activity by behavioral states. *In vivo* studies confirm that when animals explore a novel environment, OLM neuron activity decreases. This reduction disinhibits distal dendrites, creating a temporal window for synergistic CA3 and EC inputs to induce dendritic calcium signals (e.g., plateau potentials) and synaptic plasticity (e.g., LTP), thereby promoting the formation of new place cell representations and spatial memory remapping (Campbell et al., 2025). Conversely, in familiar environments, enhanced OLM cell activity suppresses unnecessary plasticity, contributing to the stabilization of established spatial memories. Furthermore, during contextual fear memory encoding, SOM neurons (particularly OLM cells) facilitate precise fear memory formation by inhibiting EC inputs related to the aversive stimulus, which only pass the strongest fear information (Singh and Topolnik, 2023).

Another type of SOM neurons is mainly located in the stratum pyramidale (SP) with axons thrown to the SO and SR layer. These SOM neurons receive inputs from CA3 and CA1, and project to fast-spiking interneurons, thereby producing a disinhibitory effect (Chamberland et al., 2024).

#### 4.2.3 Cholecystokinin (CCK)-expressing interneurons

Cholecystokinin-positive interneurons, especially CCK basket cells, provide another significant source of inhibition. Unlike PV basket cells, CCK basket cells typically exhibit regular-spiking or burst-spiking patterns, and their activity often alternates or complements that of PV cells, such as showing a transient “run-stop” response burst after locomotion ceases (Malhotra et al., 2025). CCK basket cells highly express cannabinoid CB1 receptors, mediating a state-dependent depolarization-induced suppression of inhibition. This allows their GABA release to be retrogradely regulated by postsynaptic neuronal activity, resulting in a flexible and plastic form of fine-grained inhibition (Tzilivaki et al., 2023; Malhotra et al., 2025). This mechanism is crucial for “sharpening” the place fields of place cells and enhancing spatial coding precision. Additionally, CCK neurons receive various neuromodulatory inputs from the brainstem and basal forebrain (e.g., serotonin, acetylcholine), enabling them to integrate information about the animal’s emotional, motivational, and arousal states, and participate in selective attention and reward-related information processing (Nguyen et al., 2024).

In addition to CCK basket cells, there are also CCK dendritic targeting cells and interneuron-selective interneurons. CCK dendritic targeting cells are located in SR/SLM layers, including Schaffer collateral-associated cells, apical dendrite targeting cells, and perforant path-associated cells. These cells regulate inputs from CA3 and EC, involving the selection and integration of different processes.

Some CCK neurons also target other interneurons (e.g., PV basket cells or SOM cells), indirectly enhancing PC excitability through a disinhibition mechanism. This disinhibition is a strategy of precise selection reinforcement, which permits the brain specially increase the excitability of some circuits or ensembles under a suppressed brain state. And this mechanism is critical for the control of attention and memory recollection.

Functionally, the CCK neurons in the ventral hippocampus are essential for episodic memory (Nguyen et al., 2024). These cells occupied around half of the PCs’ local inputs, and a pharmacological experiment indicated that they have feed-forward inhibition on PCs. Inhibiting these neurons during exploration, animals would more prefer reward context. In our unpublished work, we found that inhibition of CCK neurons in the dorsal hippocampus which receive direct input from the PER, could increase the discrimination rate of context fear memory. These evidence indicate that the CCK neurons are involved in reward cues and promoted selective attention (Malhotra et al., 2025).

#### 4.2.4 Vasoactive intestinal peptide (VIP)-expressing interneurons

Vasoactive intestinal peptide-positive interneurons are mainly located in SR and SLM layers, and regulate the dendritic computation within these layers. These VIP INs primarily function by inhibiting other inhibitory interneurons (e.g., PV and SOM cells), thereby indirectly enhancing pyramidal cell excitability via disinhibition (Bilash et al., 2022). This disinhibitory mechanism is particularly important during information encoding. Research shows that VIP neuron activity increases significantly when animals encounter novel contexts or during novel object recognition tasks, inhibiting VIP neurons impairs the encoding of novel information and learning (Turi et al., 2019; Tamboli et al., 2024). The activation of VIP neurons is believed to lift the inhibition imposed by PV/SOM neurons on PCs, creating favorable conditions for synaptic plasticity and the formation of new memory representations. Moreover, a subset of VIP neurons are long-range projection cells, sending axons to areas like the subiculum or entorhinal cortex, potentially involved in coordinating network activity between the hippocampus and adjacent cortical areas (Tzilivaki et al., 2023).

Ultimately, distinct class of inhibitory interneurons in the hippocampal CA1 region orchestrate the input integration and network dynamics of PCs with remarkable precision through their specific anatomical targeting, firing properties, and regulatory mechanisms, thereby playing complementary and critical roles in memory encoding, consolidation, and retrieval. PV INs, such as basket cells and chandelier cells, provide fast and powerful perisomatic inhibition. They are pivotal for generating and synchronizing network oscillations and precisely controlling the output timing of PCs. SOMs specifically regulate distal dendritic inputs from the EC via feedback inhibitory loops. And enhanced OLM activity in familiar environments contributes to memory stabilization, whereas its suppression in novel environments disinhibits distal dendrites, creating a temporal window for synergistic CA3 and EC inputs to induce plasticity and support spatial memory remapping. CCK INs exhibit a highly plastic and state-dependent form of inhibition regulated retrograde by the endocannabinoid system. They are involved in sharpening place fields of place cells and integrating information related to reward, attention, and affective states. A subset of CCK neurons indirectly enhances PC excitability via disinhibition, which is crucial for episodic memory and selective attention. VIP INs primarily exert disinhibition by suppressing PV and SOM interneurons. Their activity markedly increases in novel contexts, thereby creating permissive conditions for synaptic plasticity and the formation of new memory representations. Collectively, these functionally diverse interneuron populations form a dynamic microcircuit regulatory network operating across associative formation, ensuring the precision, efficiency, and adaptability of information processing in the hippocampus.

## 5 Discussion

Traditionally, the perirhinal cortex has been viewed as a sensory gate that relays object feature information to the hippocampus. However, accumulating evidence suggests that the PER is not a passive relay station but an active hub for associative formation and representational integration. Central to this view is the powerful inhibitory microcircuitry within the PER, particularly the inhibitory system dominated by CR neurons. This system provides the essential computational foundation for encoding highly specific sensory features and forming precisely defined associations against a noisy background. Evidence supporting this perspective is multifaceted. First, beyond processing object features, PER neuronal activity encodes associations among multimodal information, including spatial context, temporal context, and reward valence. Second, dense bidirectional anatomical connections exist between the PER and HPC, facilitating real-time, dynamic information exchange during memory encoding, rather than unidirectional transmission. Therefore, in contrast to simple feature filtering, PER serves as a critical node for long-term memory storage, preserving high-fidelity details of episodic memories.

The bidirectional dialog between the PER and HPC is fundamental to association formation. Anatomically, these regions are tightly interconnected via direct and indirect pathways. Functionally, lesions in either structure led to significant impairments in the mnemonic functions associated with the other. *In vivo* electrophysiological recordings further reveal phase synchronization between the PER and HPC in the theta and gamma frequency bands, a mechanism thought to underlie efficient information exchange. However, there’re still questions that remain to totally untangle the information flow between the PER and HPC, such as the direction of flow across distinct memory stages of behavior task, and the cells involved in these processes. Thus, techniques with higher spatiotemporal resolution are needed for future research.

Moreover, higher-order regions, including the prefrontal cortex, can top-down modulate the synchrony within the PER-HPC network, thereby mediating cognitive control over memory processes. And the neuromodulators are also pivotal in function regulation of PER-HPC network. It’s essential to situate the PER-HPC network within a whole-brain system to investigate how it supports advanced functions like emotional modulation and cognitive control of memory.

Following processing by PER, the subsequent EC, DG, and CA3 region in the indirect pathway are all deeply involved in the formation of episodic memory. Any abnormality in key regions along this main pathway can disrupt the establishment of the episodic memory framework. For instance, overactivation of the LEC–CA3 pathway impairs animals’ novel object recognition ability (Maurer et al., 2017); similarly, hyperexcitability in DG and CA3 also compromises this capacity (Johnson et al., 2018). It is noteworthy that as the similarity between objects to be discriminated increases, the expression of c-Fos in DG and CA3 rises accordingly, accompanied by enhanced activation of inhibitory neurons (Araujo et al., 2025). This indicates that novel object recognition does not rely solely on feature coding by PER or sparse coding by DG, but rather depends on coordinated and normal neural activity within the hippocampal network. In contrast, the recognition of familiar objects does not require hippocampal involvement and can be supported solely by local circuits within the perirhinal cortex.

The CA1 as the final integrator, receiving and processing inputs from the PER and other cortical areas. Different types of information (e.g., object, space, context) converge and are bound within distinct hippocampal subfields (e.g., CA1, CA3), ultimately forming a spatiotemporally specific index for episodic memories. In this integration process, diverse inhibitory interneurons within the hippocampus play crucial roles. For instance, PV basket cells provide strong feedback inhibition, sharpening the firing time windows of neuronal populations to enhance the signal-to-noise ratio and coding specificity. Other interneurons, such as those CCK neurons, may dynamically modulate the integrating information related to reward, attention, and affective states. It is precisely this refined microcircuit regulation that enables the HPC to flexibly compare internal memory representations with external sensory inputs, coordinating the encoding, consolidation, and retrieval of memories.

PER maintains bidirectional anatomical connections with both the dorsal and ventral hippocampus, yet exhibits functional specialization in the direction of information flow (Agster and Burwell, 2013). In the dorsal hippocampal (dHPC) pathway, information primarily projects from PER to dHPC (Shi and Cassell, 1999), conveying high-level object feature information (e.g., shape, texture, identity) to support the integration with specific spatial cues and construct episodic associative memories, such as object–location bindings. For example, during exploration of a novel environment, PER neurons sencode the features of objects, while dHPC neurons integrate this information with context coordinates to form a unified representation like “object A is located in context B” (Barker and Warburton, 2020).

Conversely, in the ventral hippocampal (vHPC) pathway, the information flow is predominantly from the ventral hippocampus to PER, transmitting emotional and value-related information (e.g., reward, threat) to jointly establish memories linking objects to affective and motivational significance (Agster and Burwell, 2013). For example, in conditioned fear paradigms, vHPC might transmit shock-related emotional signals to PER, enabling the association of fear with specific objects (Herry et al., 2008; Orsini et al., 2011; Sotres-Bayon et al., 2012). Similarly, in reward-based learning tasks, reward information encoded by vHPC modulates the value representation of relevant objects within PER, highlighting the role of this pathway in affective memory (Wan et al., 1999; Murray and Richmond, 2001; Sotres-Bayon et al., 2012). The core role of PER in associative memory and information integration has been substantiated across multiple species. Nevertheless, cross-species generalizations face notable limitations. First, rodent PER primarily mediates integration at the sensory level, whereas primate PER may engage more abstract cross-modal semantic networks (Suzuki and Naya, 2014). Second, functional lateralization of PER in humans remains ambiguous in animal models (Wagner et al., 2009; Ren et al., 2023). Third, interspecies differences in anatomical connectivity likely modulate PER’s regulatory mechanisms within memory networks. Future investigation should integrate cross-species computational modeling with high-resolution circuit tracing to delineate the evolutionary trajectory of PER from basic associative binding to advanced cognitive integration.

The coordinated operation of the PER-HPC circuit holds significant clinical implications. For example, in Alzheimer’s disease, gray matter atrophy and pathological changes in the PER often precede noticeable hippocampal involvement and clinical symptoms, positioning it as a potential early biomarker. Understanding the normal and pathological mechanisms of this circuit may open new avenues for intervening in memory disorders. By comparing emerging network neuromodulation and brain-computer interface technologies, scientists could explore precise interventions targeting specific circuits may yield novel therapeutic strategies for pathological memory impairments.

## References

[B1] Aggleton J. P. Brown M. W. (2005). Contrasting hippocampal and perirhinalcortex function using immediate early gene imaging. *Q. J. Exp. Psychol. Sect. B* 58 218–233. 10.1080/02724990444000131 16194966

[B2] Aggleton J. P. Kyd R. J. Bilkey D. K. (2004). When is the perirhinal cortex necessary for the performance of spatial memory tasks? *Neurosci. Biobehav. Rev.* 28 611–624. 10.1016/j.neubiorev.2004.08.007 15527866

[B3] Agster K. L. Burwell R. D. (2013). Hippocampal and subicular efferents and afferents of the perirhinal, postrhinal, and entorhinal cortices of the rat. *Behav. Brain Res.* 254 50–64. 10.1016/j.bbr.2013.07.005 23872326 PMC3792719

[B4] Ahn J.-R. Lee I. (2015). Neural correlates of object-associated choice behavior in the perirhinal cortex of rats. *J. Neurosci.* 35 1692–1705. 10.1523/jneurosci.3160-14.2015 25632144 PMC4308609

[B5] Aimone J. B. Deng W. Gage F. H. (2010). Adult neurogenesis: Integrating theories and separating functions. *Trends Cogn. Sci.* 14 325–337. 10.1016/j.tics.2010.04.003 20471301 PMC2904863

[B6] Albasser M. M. Amin E. Iordanova M. D. Brown M. W. Pearce J. M. Aggleton J. P. (2011). Separate but interacting recognition memory systems for different senses: The role of the rat perirhinal cortex. *Learn. Mem.* 18 435–443. 10.1101/lm.2132911 21685150 PMC3125609

[B7] Albasser M. M. Olarte-Sánchez C. M. Amin E. Brown M. W. Kinnavane L. Aggleton J. P. (2015). Perirhinal cortex lesions in rats: Novelty detection and sensitivity to interference. *Behav. Neurosci.* 129 227–243. 10.1037/bne0000049 26030425 PMC4450885

[B8] Albasser M. M. Poirier G. L. Aggleton J. P. (2009). Qualitatively different modes of perirhinal–hippocampal engagement when rats explore novel vs. familiar objects as revealed by c-Fos imaging. *Eur. J. Neurosci.* 31 134–147. 10.1111/j.1460-9568.2009.07042.x 20092559 PMC4235254

[B9] Allen L. M. Lesyshyn R. A. O’Dell S. J. Allen T. A. Fortin N. J. (2020). The hippocampus, prefrontal cortex, and perirhinal cortex are critical to incidental order memory. *Behav. Brain Res.* 379:112215. 10.1016/j.bbr.2019.112215 31682866 PMC6917868

[B10] Andersen P. (1990). Synaptic integration in hippocampal CA1 pyramids. *Prog. Brain Res.* 1990 215–222. 10.1016/s0079-6123(08)61251-0 2168057

[B11] Araujo A. P. C. Parente J. S. Coutinho S. L. O. Castelo-Branco R. Meurer Y. S. R. Barbosa F. F. (2025). Between similarity and difference: Network dynamics of the hippocampal- parahippocampal circuitry in pattern separation of male Wistar rats. *Front. Cell. Neurosci.* 19:1648536. 10.3389/fncel.2025.1648536 41342014 PMC12669181

[B12] Balderas I. Rodriguez-Ortiz C. J. Bermudez-Rattoni F. (2013). Retrieval and reconsolidation of object recognition memory are independent processes in the perirhinal cortex. *Neuroscience* 253 398–405. 10.1016/j.neuroscience.2013.09.001 24042035

[B13] Barinka F. Salaj M. Rybar J. Krajcovicova E. Kubova H. Druga R. (2012). Calretinin, parvalbumin and calbindin immunoreactive interneurons in perirhinal cortex and temporal area Te3V of the rat brain: Qualitative and quantitative analyses. *Brain Res.* 1436 68–80. 10.1016/j.brainres.2011.12.014 22221733

[B14] Barker G. R. I. Warburton E. C. (2020). Putting objects in context: A prefrontal-hippocampal-perirhinal cortex network. *Brain Neurosci. Adv.* 4:2398212820937621. 10.1177/2398212820937621 32954004 PMC7479864

[B15] Bartko S. J. Winters B. D. Cowell R. A. Saksida L. M. Bussey T. J. (2007). Perceptual functions of perirhinal cortex in rats: Zero-delay object recognition and simultaneous oddity discriminations. *J. Neurosci.* 27 2548–2559. 10.1523/JNEUROSCI.5171-06.2007 17344392 PMC6672512

[B16] Bartley T. D. Furtak S. C. (2021). Perirhinal damage produces modality-dependent deficits in fear learning. *Neurobiol. Learn Mem.* 181:107427. 10.1016/j.nlm.2021.107427 33798696

[B17] Beaudin S. A. Singh T. Agster K. L. Burwell R. D. (2013). Borders and comparative cytoarchitecture of the perirhinal and postrhinal cortices in an F1 hybrid mouse. *Cereb. Cortex* 23 460–476. 10.1093/cercor/bhs038 22368084 PMC3584955

[B18] Bedwell S. A. Billett E. E. Crofts J. J. MacDonald D. M. Tinsley C. J. (2015). The topology of connections between rat prefrontal and temporal cortices. *Front. Syst. Neurosci.* 9:80. 10.3389/fnsys.2015.00080 26042005 PMC4438597

[B19] Biella G. Uva L. de Curtis M. (2002). Propagation of neuronal activity along the neocortical-perirhinal-entorhinal pathway in the guinea pig. *J. Neurosci.* 22 9972–9979. 10.1523/JNEUROSCI.22-22-09972.2002 12427854 PMC6757852

[B20] Bilash O. M. Chavlis S. Poirazi P. Basu J. (2022). Lateral entorhinal cortex inputs modulate hippocampal dendritic excitability by recruiting a local disinhibitory microcircuit. *Cell* [Preprint]. 10.1101/2022.01.13.476247 PMC10337264 36640337

[B21] Binini N. Talpo F. Spaiani P. Maniezzo C. Pedrazzoli M. Raffin F. et al. (2021). Membrane resonance in pyramidal and GABAergic neurons of the mouse perirhinal cortex. *Front. Cell. Neurosci.* 15:703407. 10.3389/fncel.2021.703407 34366789 PMC8339929

[B22] Bos J. J. Vinck M. van Mourik-Donga L. A. Jackson J. C. Witter M. P. Pennartz C. M. A. (2017). Perirhinal firing patterns are sustained across large spatial segments of the task environment. *Nat. Commun.* 8:15602. 10.1038/ncomms15602 28548084 PMC5458559

[B23] Brown M. W. Aggleton J. P. (2001). Recognition memory: What are the roles of the perirhinal cortex and hippocampus? *Nat. Rev. Neurosci.* 2 51–61. 10.1038/35049064 11253359

[B24] Burke S. N. Gaynor L. S. Barnes C. A. Bauer R. M. Bizon J. L. Roberson E. D. et al. (2018). Shared functions of perirhinal and parahippocampal cortices: Implications for cognitive aging. *Trends Neurosci.* 41 349–359. 10.1016/j.tins.2018.03.001 29555181 PMC5970964

[B25] Burwell R. D. (2006). The parahippocampal region: Corticocortical connectivity. *Ann. N. Y. Acad. Sci.* 911 25–42. 10.1111/j.1749-6632.2000.tb06717.x 10911865

[B26] Burwell R. D. (2024). The anatomy of context. *Hippocampus* 35:e23668. 10.1002/hipo.23668 39721972

[B27] Burwell R. D. Amaral D. G. (1998a). Cortical afferents of the perirhinal, postrhinal, and entorhinal cortices of the rat. *J. Comp. Neurol.* 398 179–205. 10.1002/(sici)1096-9861(19980824)398:2<179:aid-cne3<3.0.co;2-y 9700566

[B28] Burwell R. D. Amaral D. G. (1998b). Perirhinal and postrhinal cortices of the rat: Interconnectivity and connections with the entorhinal cortex. *J. Comp. Neurol.* 391 293–321. 10.1002/(sici)1096-9861(19980216)391:3<293::aid-cne2<3.0.co;2-x 9492202

[B29] Burwell R. D. Witter M. P. Amaral D. G. (1995). Perirhinal and postrhinal cortices of the rat: A review of the neuroanatomical literature and comparison with findings from the monkey brain. *Hippocampus* 5 390–408. 10.1002/hipo.450050503 8773253

[B30] Camillo D. Ahmadlou M. Saiepour M. H. Yasaminshirazi M. Levelt C. N. Heimel J. A. (2018). Visual processing by calretinin expressing inhibitory neurons in mouse primary visual cortex. *Sci Rep.* 8:12355. 10.1038/s41598-018-30958-w 30120412 PMC6098074

[B31] Campbell E. P. Martin L. Magee J. C. Grienberger C. (2025). Dendrite-targeting OLM interneurons regulate the formation of learning-related CA1 place cell representations. *bioRxiv* [Preprint]. 10.64898/2025.12.21.695825 41509422 PMC12776205

[B32] Carr M. F. Frank L. M. (2012). A single microcircuit with multiple functions: State dependent information processing in the hippocampus. *Curr. Opin. Neurobiol.* 22 704–708. 10.1016/j.conb.2012.03.007 22480878 PMC3438355

[B33] Chamberland S. Grant G. Machold R. Nebet E. R. Tian G. Stich J. et al. (2024). Functional specialization of hippocampal somatostatin- expressing interneurons. *Proc. Natl. Acad. Sci. U.S.A.* 121:e2306382121. 10.1073/pnas.2306382121 38640347 PMC11047068

[B34] Chao O. Y. de Souza Silva M. A. Yang Y. M. Huston J. P. (2020). The medial prefrontal cortex - hippocampus circuit that integrates information of object, place and time to construct episodic memory in rodents: Behavioral, anatomical and neurochemical properties. *Neurosci. Biobehav. Rev.* 113 373–407. 10.1016/j.neubiorev.2020.04.007 32298711 PMC7302494

[B35] Chen H. Naya Y. (2020). Forward processing of object–location association from the ventral stream to medial temporal lobe in nonhuman primates. *Cereb. Cortex* 30 1260–1271. 10.1093/cercor/bhz164 31408097

[B36] Chen H. Zhou W. Yang J. (2019). Dissociation of the perirhinal cortex and hippocampus during discriminative learning of similar objects. *J. Neurosci.* 39 6190–6201. 10.1523/JNEUROSCI.3181-18.2019 31167939 PMC6668204

[B37] Chen S. Cheng N. Chen X. Wang C. (2024). Integration and competition between space and time in the hippocampus. *Neuron* 112 3651–3664.e8. 10.1016/j.neuron.2024.08.007 39241779

[B38] Clark A. M. Bouret S. Young A. M. Murray E. A. Richmond B. J. (2013). Interaction between orbital prefrontal and Rhinal cortex is required for normal estimates of expected value. *J. Neurosci.* 33 1833–1845. 10.1523/jneurosci.3605-12.2013 23365223 PMC3725547

[B39] de Curtis M. Paré D. (2004). The rhinal cortices: A wall of inhibition between the neocortex and the hippocampus. *Prog. Neurobiol.* 74 101–110. 10.1016/j.pneurobio.2004.08.005 15518955

[B40] de Villers-Sidani E. Tahvildari B. Alonso A. (2004). Synaptic activation patterns of the perirhinal-entorhinal inter-connections. *Neuroscience* 129 255–265. 10.1016/j.neuroscience.2004.07.044 15489047

[B41] Deacon T. W. Eichenbaum H. Rosenberg P. Eckmann K. W. (1983). Afferent connections of the perirhinal cortex in the rat. *J. Comp. Neurol*. 220 168–190. 10.1002/cne.902200205 6643724

[B42] Dorman R. Bos J. J. Vinck M. A. Marchesi P. Fiorilli J. Lorteije J. A. M. et al. (2023). Spike-based coupling between single neurons and populations across rat sensory cortices, perirhinal cortex, and hippocampus. *Cereb. Cortex* 33 8247–8264. 10.1093/cercor/bhad111 37118890 PMC10425201

[B43] Dudok B. Szoboszlay M. Paul A. Klein P. M. Liao Z. Hwaun E. et al. (2021). Recruitment and inhibitory action of hippocampal axo-axonic cells during behavior. *Neuron* 109 3838–3850.e8. 10.1016/j.neuron.2021.09.033 34648750 PMC8639676

[B44] Eichenbaum H. (2017). Prefrontal–hippocampal interactions in episodic memory. *Nat. Rev. Neurosci.* 18 547–558. 10.1038/nrn.2017.74 28655882

[B45] Eradath M. K. Mogami T. Wang G. Tanaka K. (2015). Time context of cue-outcome associations represented by neurons in perirhinal cortex. *J. Neurosci.* 35 4350–4365. 10.1523/JNEUROSCI.4730-14.2015 25762680 PMC4355203

[B46] Feinberg L. M. Allen T. A. Ly D. Fortin N. J. (2012). Recognition memory for social and non-social odors: Differential effects of neurotoxic lesions to the hippocampus and perirhinal cortex. *Neurobiol. Learn. Mem.* 97 7–16. 10.1016/j.nlm.2011.08.008 21930227

[B47] Fiorilli J. Bos J. J. Grande X. Lim J. Düzel E. Pennartz C. M. A. (2021). Reconciling the object and spatial processing views of the perirhinal cortex through task-relevant unitization. *Hippocampus* 31 737–755. 10.1002/hipo.23304 33523577 PMC8359385

[B48] Fiorilli J. Marchesi P. Ruikes T. Huis in ’t Veld G. Buckton R. Quintero M. D. et al. (2024). Neural correlates of object identity and reward outcome in the sensory cortical-hippocampal hierarchy: Coding of motivational information in perirhinal cortex. *Cereb. Cortex* 34:bhae002. 10.1093/cercor/bhae002 38314581 PMC10847907

[B49] Francavilla R. Luo X. Magnin E. Tyan L. Topolnik L. (2015). Coordination of dendritic inhibition through local disinhibitory circuits. *Front. Synaptic Neurosci.* 7:5. 10.3389/fnsyn.2015.00005 25767448 PMC4341546

[B50] Fuchsberger T. Paulsen O. (2022). Modulation of hippocampal plasticity in learning and memory. *Curr. Opin. Neurobiol.* 75:102558. 10.1016/j.conb.2022.102558 35660989

[B51] Fujimichi R. Naya Y. Koyano K. W. Takeda M. Takeuchi D. Miyashita Y. (2010). Unitized representation of paired objects in area 35 of the macaque perirhinal cortex. *Eur. J. Neurosci.* 32 659–667. 10.1111/j.1460-9568.2010.07320.x 20718858

[B52] Furtak S. C. Wei S.-M. Agster K. L. Burwell R. D. (2007). Functional neuroanatomy of the parahippocampal region in the rat: The perirhinal and postrhinal cortices. *Hippocampus* 17 709–722. 10.1002/hipo.20314 17604355

[B53] Guet-McCreight A. Skinner F. K. Topolnik L. (2020). common principles in functional organization of VIP/calretinin cell-driven disinhibitory circuits across cortical areas. *Front. Neural Circuits* 14:32. 10.3389/fncir.2020.00032 32581726 PMC7296096

[B54] Hainmueller T. Cazala A. Huang L.-W. Bartos M. (2024). Subfield-specific interneuron circuits govern the hippocampal response to novelty in male mice. *Nat. Commun.* 15:714. 10.1038/s41467-024-44882-3 38267409 PMC10808551

[B55] Harris Bozer A. L. Uhelski M. L. Li A. L. (2017). Extrapolating meaning from local field potential recordings. *J. Integr. Neurosci.* 16 107–126. 10.3233/JIN-170011 28891502

[B56] Headley D. B. Kanta V. Paré D. (2017). Intra- and interregional cortical interactions related to sharp-wave ripples and dentate spikes. *J. Neurophysiol.* 117 556–565. 10.1152/jn.00644.2016 27832604 PMC5288472

[B57] Hernandez A. R. Reasor J. E. Truckenbrod L. M. Lubke K. N. Johnson S. A. Bizon J. L. et al. (2017). Medial prefrontal-perirhinal cortical communication is necessary for flexible response selection. *Neurobiol. Learn. Mem.* 137 36–47. 10.1016/j.nlm.2016.10.012 27815215 PMC5214530

[B58] Herry C. Ciocchi S. Senn V. Demmou L. Muller C. Luthi A. (2008). Switching on and off fear by distinct neuronal circuits. *Nature* 454 600–606. 10.1038/nature07166 18615015

[B59] Holdstock J. S. Hocking J. Notley P. Devlin J. T. Price C. J. (2009). Integrating visual and tactile information in the perirhinal cortex. *Cereb. Cortex* 19 2993–3000. 10.1093/cercor/bhp073 19386635 PMC2774401

[B60] Huang C.-C. Rolls E. T. Hsu C.-C. H. Feng J. Lin C.-P. (2021). Extensive cortical connectivity of the human hippocampal memory system: Beyond the “What” and “Where” dual stream model. *Cereb. Cortex* 31 4652–4669. 10.1093/cercor/bhab113 34013342 PMC8866812

[B61] Huang T.-H. Lin Y.-S. Hsiao C.-W. Wang L.-Y. Ajibola M. I. Abdulmajeed W. I. et al. (2023). Differential expression of GABAA receptor subunits δ and α6 mediates tonic inhibition in parvalbumin and somatostatin interneurons in the mouse hippocampus. *Front. Cell. Neurosci.* 17:1146278. 10.3389/fncel.2023.1146278 37545878 PMC10397515

[B62] Hwang E. Willis B. S. Burwell R. D. (2018). Prefrontal connections of the perirhinal and postrhinal cortices in the rat. *Behav. Brain Res.* 354 8–21. 10.1016/j.bbr.2017.07.032 28765070 PMC6087504

[B63] Jacklin D. L. Cloke J. M. Potvin A. Garrett I. Winters B. D. (2016). The dynamic multisensory engram: Neural circuitry underlying crossmodal object recognition in rats changes with the nature of object experience. *J. Neurosci.* 36 1273–1289. 10.1523/JNEUROSCI.3043-15.2016 26818515 PMC6604816

[B64] Jayachandran M. Linley S. B. Schlecht M. Mahler S. V. Vertes R. P. Allen T. A. (2019). Prefrontal pathways provide top-down control of memory for sequences of events. *Cell Rep.* 28 640–654.e6. 10.1016/j.celrep.2019.06.053 31315044 PMC6662648

[B65] Jeong N. Singer A. C. (2022). Learning from inhibition: Functional roles of hippocampal CA1 inhibition in spatial learning and memory. *Curr. Opin. Neurobiol.* 76:102604. 10.1016/j.conb.2022.102604 35810533 PMC11414469

[B66] Jo Y. S. Lee I. (2010). Disconnection of the hippocampal-perirhinal cortical circuits severely disrupts object-place paired associative memory. *J. Neurosci.* 30 9850–9858. 10.1523/JNEUROSCI.1580-10.2010 20660267 PMC2913067

[B67] Johnson S. A. Turner S. M. Lubke K. N. Cooper T. L. Fertal K. E. Bizon J. L. et al. (2018). Experience-dependent effects of muscimol-induced hippocampal excitation on mnemonic discrimination. *Front. Syst. Neurosci.* 12:72. 10.3389/fnsys.2018.00072 30687032 PMC6335355

[B68] Kajiwara R. Tominaga T. (2021). Perirhinal cortex area 35 controls the functional link between the perirhinal and entorhinal-hippocampal circuitry: D-type potassium channel-mediated gating of neural propagation from the perirhinal cortex to the entorhinal-hippocampal circuitry. *Bioessays* 43:e2000084. 10.1002/bies.202000084 33236360

[B69] Katz Y. Menon V. Nicholson D. A. Geinisman Y. Kath W. L. Spruston N. (2009). Synapse distribution suggests a two-stage model of dendritic integration in CA1 pyramidal neurons. *Neuron* 63 171–177. 10.1016/j.neuron.2009.06.023 19640476 PMC2921807

[B70] Kealy J. Commins S. (2011). The rat perirhinal cortex: A review of anatomy, physiology, plasticity, and function. *Prog. Neurobiol.* 93 522–548. 10.1016/j.pneurobio.2011.03.002 21420466

[B71] Keene C. S. Bladon J. McKenzie S. Liu C. D. O’Keefe J. Eichenbaum H. (2016). Complementary functional organization of neuronal activity patterns in the perirhinal, lateral entorhinal, and medial entorhinal cortices. *J. Neurosci.* 36 3660–3675. 10.1523/JNEUROSCI.4368-15.2016 27030753 PMC4812128

[B72] Kinnavane L. Amin E. Olarte-Sánchez C. M. Aggleton J. P. (2016). Detecting and discriminating novel objects: The impact of perirhinal cortex disconnection on hippocampal activity patterns. *Hippocampus* 26 1393–1413. 10.1002/hipo.22615 27398938 PMC5082501

[B73] Kosel K. C. Van Hoesen G. W. Rosene D. L. (1983). A direct projection from the perirhinal cortex (area 35) to the subiculum in the rat. *Brain* 269 347–351. 10.1016/0006-8993(83)90144-0 6883086

[B74] Kotak V. C. Mirallave A. Mowery T. M. Sanes D. H. (2017). GABAergic inhibition gates excitatory LTP in perirhinal cortex. *Hippocampus* 27 1217–1223. 10.1002/hipo.22799 28881444 PMC5745066

[B75] Kreher M. A. Johnson S. A. Mizell J.-M. Chetram D. K. Guenther D. T. Lovett S. D. et al. (2019). The perirhinal cortex supports spatial intertemporal choice stability. *Neurobiol. Learn. Mem.* 162 36–46. 10.1016/j.nlm.2019.05.002 31125611 PMC6591720

[B76] Lee D. G. McLachlan C. A. Nogueira R. Kwon O. Carey A. E. House G. et al. (2024). Perirhinal cortex learns a predictive map of the task environment. *Nat. Commun.* 15:5544. 10.1038/s41467-024-47365-7 38956015 PMC11219840

[B77] Li X. Li Y. Zhang J. Zhang X. (2019). Selective targeting of perirhinal cortex projection to hippocampal CA1 interneurons. *Neurosci. Bull.* 35 763–765. 10.1007/s12264-019-00363-y 30879176 PMC6617490

[B78] Liguz-Lecznar M. Urban-Ciecko J. Kossut M. (2016). Somatostatin and somatostatin-containing neurons in shaping neuronal activity and plasticity. *Front. Neural Circuits* 10:48. 10.3389/fncir.2016.00048 27445703 PMC4927943

[B79] Lim H.-Y. Lee I. (2024). Subpopulations of neurons in the perirhinal cortex enable both modality-specific and modality-invariant recognition of objects. *PLoS Biol.* 22:e3002713. 10.1371/journal.pbio.3002713 38924050 PMC11233021

[B80] Lisman J. E. Jensen O. (2013). The theta-gamma neural code. *Neuron* 77 1002–1016. 10.1016/j.neuron.2013.03.007 23522038 PMC3648857

[B81] Liu Z. Murray E. A. Richmond B. J. (2000). Learning motivational significance of visual cues for reward schedules requires rhinal cortex. *Nat. Neurosci*. 3 1307–1315. 10.1038/81841 11100152

[B82] Luo W. Yun D. Hu Y. Tian M. Yang J. Xu Y. et al. (2022). Acquiring new memories in neocortex of hippocampal-lesioned mice. *Nat. Commun.* 13:1601. 10.1038/s41467-022-29208-5 35332120 PMC8948206

[B83] Malhotra S. Donneger F. Farrell J. S. Dudok B. Losonczy A. Soltesz I. (2025). Integrating endocannabinoid signaling, CCK interneurons, and hippocampal circuit dynamics in behaving animals. *Neuron* 113 1862–1885. 10.1016/j.neuron.2025.03.016 40267911 PMC12410895

[B84] Martina M. Royer S. Paré D. (2001). Cell-type-specific GABA responses and chloride homeostasis in the cortex and amygdala. *J. Neurophysiol.* 86 2887–2895. 10.1152/jn.2001.86.6.2887 11731545

[B85] Maurer A. P. Johnson S. A. Hernandez A. R. Reasor J. Cossio D. M. Fertal K. E. et al. (2017). Age-related changes in lateral entorhinal and CA3 neuron allocation predict poor performance on object discrimination. *Front. Syst. Neurosci.* 11:49. 10.3389/fnsys.2017.00049 28713251 PMC5491840

[B86] McLachlan C. A. Lee D. G. Kwon O. Delgado K. M. Manjrekar N. Yao Z. et al. (2025). Transcriptional determinants of goal-directed learning and representational drift in the parahippocampal cortex. *Cell Rep.* 44:115175. 10.1016/j.celrep.2024.115175 39792551 PMC11920904

[B87] Medalla M. Mo B. Nasar R. Zhou Y. Park J. Luebke J. I. (2023). Comparative features of calretinin, calbindin, and parvalbumin expressing interneurons in mouse and monkey primary visual and frontal cortices. *J. Comp. Neurol.* 531 1934–1962. 10.1002/cne.25514 37357562 PMC10749991

[B88] Messinger A. Squire L. R. Zola S. M. Albright T. D. (2001). Neuronal representations of stimulus associations develop in the temporal lobe during learning. *Proc. Natl. Acad. Sci. U.S.A.* 98 12239–12244. 10.1073/pnas.211431098 11572946 PMC59829

[B89] Miyashita Y. (2019). Perirhinal circuits for memory processing. *Nat. Rev. Neurosci.* 20 577–592. 10.1038/s41583-019-0213-6 31485007

[B90] Morici J. F. Cicuttin G. Silva A. Gallo F. T. Miranda M. Belluscio M. et al. (2022a). Serotonin type 2a receptor in the prefrontal cortex controls perirhinal cortex excitability during object recognition memory recall. *Neuroscience* 497 196–205. 10.1016/j.neuroscience.2022.05.015 35597334

[B91] Morici J. F. Miranda M. Gallo F. T. Zanoni B. Bekinschtein P. Weisstaub N. V. (2018). 5-HT2a receptor in mPFC influences context-guided reconsolidation of object memory in perirhinal cortex. *eLife* 7:e33746. 10.7554/eLife.33746 29717980 PMC5931799

[B92] Morici J. F. Weisstaub N. V. Zold C. L. (2022b). Hippocampal-medial prefrontal cortex network dynamics predict performance during retrieval in a context-guided object memory task. *Proc. Natl. Acad. Sci. U.S.A.* 119 e2203024119. 10.1073/pnas.2203024119 35561217 PMC9171913

[B93] Murray E. A. Richmond B. J. (2001). Role of perirhinal cortex in object perception, memory, and associations. *Cogn. Neurosci*. 11 188–193. 10.1016/s0959-4388(00)00195-1 11301238

[B94] Naber P. A. Witter M. P. Lopes da Silva F. H. (1999). Perirhinal cortex input to the hippocampus in the rat: Evidence for parallel pathways, both direct and indirect. A combined physiological and anatomical study. *Eur J. Neurosci.* 11 4119–4133. 10.1046/j.1460-9568.1999.00835.x 10583500

[B95] Naya Y. (2016). Declarative association in the perirhinal cortex. *Neurosci. Res.* 113 12–18. 10.1016/j.neures.2016.07.001 27418578

[B96] Naya Y. Chen H. Yang C. Suzuki W. A. (2017). Contributions of primate prefrontal cortex and medial temporal lobe to temporal-order memory. *Proc. Natl. Acad. Sci. U.S.A.* 114 13555–13560. 10.1073/pnas.1712711114 29192021 PMC5754788

[B97] Naya Y. Yoshida M. Miyashita Y. (2003). Forward processing of long-term associative memory in monkey inferotemporal cortex. *J. Neurosci.* 23 2861–2871. 10.1523/JNEUROSCI.23-07 12684473 PMC6742072

[B98] Nguyen R. Sivakumaran S. Lambe E. K. Kim J. C. (2024). Ventral hippocampal cholecystokinin interneurons gate contextual reward memory. *iScience* 27:108824. 10.1016/j.isci.2024.108824 38303709 PMC10831933

[B99] Norman G. Eacott M. J. (2004). Impaired object recognition with increasing levels of feature ambiguity in rats with perirhinal cortex lesions. *Behav. Brain Res.* 148 79–91. 10.1016/s0166-4328(03)00176-1 14684250

[B100] Obermayer J. Luchicchi A. Heistek T. S. de Kloet S. F. Terra H. Bruinsma B. et al. (2019). Prefrontal cortical ChAT-VIP interneurons provide local excitation by cholinergic synaptic transmission and control attention. *Nat. Commun.* 10:5280. 10.1038/s41467-019-13244-9 31754098 PMC6872593

[B101] Ohyama K. Sugase-Miyamoto Y. Matsumoto N. Shidara M. Sato C. (2012). Stimulus-related activity during conditional associations in monkey perirhinal cortex neurons depends on upcoming reward outcome. *J. Neurosci.* 32 17407–17419. 10.1523/jneurosci.2878-12.2012 23197732 PMC6621831

[B102] Olarte-Sánchez C. M. Amin E. Warburton E. C. Aggleton J. P. (2015). Perirhinal cortex lesions impair tests of object recognition memory but spare novelty detection. *Eur. J. Neurosci.* 42 3117–3127. 10.1111/ejn.13106 26474445 PMC4737320

[B103] Orsini C. A. Kim J. H. Knapska E. Maren S. (2011). Hippocampal and prefrontal projections to the basal amygdala mediate contextual regulation of fear after extinction. *J. Neurosci.* 31 17269–17277. 10.1523/JNEUROSCI.4095-11.2011 22114293 PMC3241946

[B104] Pelkey K. A. Chittajallu R. Craig M. T. Tricoire L. Wester J. C. McBain C. J. (2017). Hippocampal GABAergic inhibitory interneurons. *Physiol. Rev.* 97 1619–1747. 10.1152/physrev.00007.2017 28954853 PMC6151493

[B105] Pelletier J. G. Apergis J. Paré D. (2004). Low-probability transmission of neocortical and entorhinal impulses through the perirhinal cortex. *J. Neurophysiol.* 91 2079–2089. 10.1152/jn.01197.2003 15069098

[B106] Pinto A. Fuentes C. Pare D. (2006). Feedforward inhibition regulates perirhinal transmission of neocortical inputs to the entorhinal cortex: Ultrastructural study in guinea pigs. *J. Comp. Neurol.* 495 722–734. 10.1002/cne.20905 16506192 PMC4425285

[B107] Pishdadian S. Coutrot A. Webber L. Hornberger M. Spiers H. Rosenbaum R. S. (2024). Combining patient-lesion and big data approaches to reveal hippocampal contributions to spatial memory and navigation. *iScience* 27:109977. 10.1016/j.isci.2024.109977 38947515 PMC11214368

[B108] Piskorowski R. A. Chevaleyre V. (2011). Synaptic integration by different dendritic compartments of hippocampal CA1 and CA2 pyramidal neurons. *Cell. Mol. Life Sci.* 69 75–88. 10.1007/s00018-011-0769-4 21796451 PMC11115016

[B109] Qian F. K. Li Y. Magee J. C. (2025). Mechanisms of experience-dependent place-cell referencing in hippocampal area CA1. *Nat. Neurosci.* 28 1486–1496. 10.1038/s41593-025-01930-5 40169932 PMC12229891

[B110] Qureshi O. A. Leake J. Delaney A. J. Killcross S. Westbrook R. F. Holmes N. M. (2023). Danger changes the way the brain consolidates neutral information; and does so by interacting with processes involved in the encoding of that information. *J. Neurosci.* 43 2934–2949. 10.1523/jneurosci.1796-22.2023 36927572 PMC10124951

[B111] Ren J. Huang F. Gao C. Gott J. Schoch S. F. Qin S. et al. (2023). Functional lateralization of the medial temporal lobe in novel associative processing during creativity evaluation. *Cortex* 33 1186–1206. 10.1093/cercor/bhac129 35353185 PMC9930633

[B112] Renart A. Parga N. Rolls E. T. (1999). Backward projections in the cerebral cortex: Implications for memory storage. *Neural Comput.* 11 1349–1388. 10.1162/089976699300016278 10423499

[B113] Rolls E. T. (2015). Diluted connectivity in pattern association networks facilitates the recall of information from the hippocampus to the neocortex. *Prog. Brain Res.* 219 21–43. 10.1016/bs.pbr.2015.03.007 26072232

[B114] Rolls E. T. Kesner R. P. (2016). Pattern separation and pattern completion in the hippocampal system. Introduction to the special issue. *Neurobiol. Learn. Mem.* 129 1–3. 10.1016/j.nlm.2016.02.001 26872593

[B115] Rolls E. T. Zhang C. Feng J. (2024). Hippocampal storage and recall of neocortical “What”–“Where” representations. *Hippocampus* 34 608–624. 10.1002/hipo.23636 39221708

[B116] Rolotti S. V. Ahmed M. S. Szoboszlay M. Geiller T. Negrean A. Blockus H. et al. (2022). Local feedback inhibition tightly controls rapid formation of hippocampal place fields. *Neuron* 110 783–794.e6. 10.1016/j.neuron.2021.12.003 34990571 PMC8897257

[B117] Rosen Z. B. Cheung S. Siegelbaum S. A. (2015). Midbrain dopamine neurons bidirectionally regulate CA3-CA1 synaptic drive. *Nat. Neurosci.* 18 1763–1771. 10.1038/nn.4152 26523642 PMC11186581

[B118] Salaj M. Barinka F. Kubová H. Druga R. (2021). Differences in expression of calcium binding proteins in the perirhinal and retrosplenial cortex of the rat. *Physiol. Res.* 70 273–285. 10.33549/physiolres.934548 33992048 PMC8820584

[B119] Schlichting M. L. Preston A. R. (2016). Hippocampal-medial prefrontal circuit supports memory updating during learning and post-encoding rest. *Neurobiol. Learn. Mem.* 134 91–106. 10.1016/j.nlm.2015.11.005 26608407 PMC4879117

[B120] Schneider-Mizell C. M. Bodor A. L. Brittain D. Buchanan J. Bumbarger D. J. Elabbady L. et al. (2025). Inhibitory specificity from a connectomic census of mouse visual cortex. *Nature* 640 448–458. 10.1038/s41586-024-07780-8 40205209 PMC11981935

[B121] Scoville W. B. Milner B. (1957). Loss of recent memory after bilateral hippocampal lesions. *J. Neurol. Neurosurg. Psychiatry* 20 11–21. 10.1136/jnnp.20.1.11 13406589 PMC497229

[B122] Shen W. Li Z. Tang Y. Han P. Zhu F. Dong J. et al. (2022). Somatostatin interneurons inhibit excitatory transmission mediated by astrocytic GABAB and presynaptic GABAB and adenosine A1 receptors in the hippocampus. *J. Neurochem.* 163 310–326. 10.1111/jnc.15662 35775994

[B123] Shi C.-J. Cassell M. D. (1999). Perirhinal cortex projections to the amygdaloid complex and hippocampal formation in the rat. *J. Comp. Neurol*. 406 299–328. 10.1002/(sici)1096-9861(19990412)406:3<299::aid-cne2<3.0.co;2-9 10102498

[B124] Singh S. Topolnik L. (2023). Inhibitory circuits in fear memory and fear-related disorders. *Front. Neural Circuits* 17:1122314. 10.3389/fncir.2023.1122314 37035504 PMC10076544

[B125] Sotres-Bayon F. Sierra-Mercado D. Pardilla-Delgado E. Quirk G. J. (2012). Gating of fear in prelimbic cortex by hippocampal and amygdala inputs. *Neuron* 76 804–812. 10.1016/j.neuron.2012.09.028 23177964 PMC3508462

[B126] Suter E. E. Weiss C. Disterhoft J. F. (2013). Perirhinal and postrhinal, but not lateral entorhinal, cortices are essential for acquisition of trace eyeblink conditioning. *Learn. Mem.* 20 80–84. 10.1101/lm.028894.112 23322556 PMC3549062

[B127] Suzuki W. A. Naya Y. (2014). The perirhinal cortex. *Annu. Rev. Neurosci.* 37 39–53. 10.1146/annurev-neuro-071013-014207 25032492

[B128] Tamboli S. Singh S. Topolnik D. Barkat M. E. A. Radhakrishnan R. Guet-McCreight A. et al. (2024). Mouse hippocampal CA1 VIP interneurons detect novelty in the environment and support recognition memory. *Cell Rep.* 43:114115. 10.1016/j.celrep.2024.114115 38607918

[B129] Tanaka K. Z. McHugh T. J. (2018). The Hippocampal Engram as a Memory Index. *J. Exp. Neurosci.* 12:1179069518815942. 10.1177/1179069518815942 30546263 PMC6287299

[B130] Topolnik L. Tamboli S. (2022). The role of inhibitory circuits in hippocampal memory processing. *Nat. Rev. Neurosci.* 23 476–492. 10.1038/s41583-022-00599-0 35637416

[B131] Torta A. B. L. Komorowski R. W. Manns J. R. Kopell N. J. Eichenbaum H. (2009). Theta–gamma coupling increases during the learning of item–context associations. *Proc. Natl. Acad. Sci. U.S.A.* 106 20942–20947. 10.1073/pnas.0911331106 19934062 PMC2791641

[B132] Trettel S. G. Agster K. L. Burwell R. D. (2021). Perirhinal and postrhinal damage have different consequences on attention as assessed in the five-choice serial reaction time task. *eNeuro* 8:ENEURO.0210-21.2021. 10.1523/ENEURO.0210-21.2021 34475265 PMC8462067

[B133] Turi G. F. Li W.-K. Chavlis S. Pandi I. O’Hare J. Priestley J. B. et al. (2019). Vasoactive intestinal polypeptide-expressing interneurons in the hippocampus support goal-oriented spatial learning. *Neuron* 101 1150–1165.e8. 10.1016/j.neuron.2019.01.009 30713030 PMC6428605

[B134] Tzilivaki A. Tukker J. J. Maier N. Poirazi P. Sammons R. P. Schmitz D. (2023). Hippocampal GABAergic interneurons and memory. *Neuron* 111 3154–3175. 10.1016/j.neuron.2023.06.016 37467748 PMC10593603

[B135] Udakis M. Claydon M. D. B. Zhu H. W. Oakes E. C. Mellor J. R. (2025). Hippocampal OLM interneurons regulate CA1 place cell plasticity and remapping. *Nat. Commun.* 16:9912. 10.1038/s41467-025-64859-0 41219196 PMC12606138

[B136] Vaidya S. P. Li G. Chitwood R. A. Li Y. Magee J. C. (2025). Formation of an expanding memory representation in the hippocampus. *Nat. Neurosci*. 28 1510–1518. 10.1038/s41593-025-01986-3 40467863 PMC12229897

[B137] van Strien N. M. Cappaert N. L. M. Witter M. P. (2009). The anatomy of memory: An interactive overview of the parahippocampal-hippocampal network. *Nat. Rev. Neurosci.* 10 272–282. 10.1038/nrn2614 19300446

[B138] Vilberg K. L. Davachi L. (2013). Perirhinal-hippocampal connectivity during reactivation is a marker for object-based memory consolidation. *Neuron* 79 1232–1242. 10.1016/j.neuron.2013.07.013 23993700 PMC3837480

[B139] Wagner D. D. Sziklas V. Garver K. E. Jones-Gotman M. (2009). Material-specific lateralization of working memory in the medial temporal lobe. *Neuropsychologia* 47 112–122. 10.1016/j.neuropsychologia.2008.08.010 18775736

[B140] Wan H. Aggleton J. P. Brown M. W. (1999). Different contributions of the hippocampus and perirhinal cortex to recognition memory. *J. Neurosci.* 19 1142–1148. 10.1523/JNEUROSCI.19-03-01142.1999 9920675 PMC6782155

[B141] Warburton E. C. Brown M. W. (2010). Findings from animals concerning when interactions between perirhinal cortex, hippocampus and medial prefrontal cortex are necessary for recognition memory. *Neuropsychologia* 48 2262–2272. 10.1016/j.neuropsychologia.2009.12.022 20026141

[B142] Watarai A. Tao K. Okuyama T. (2025). Representation of sex-specific social memory in ventral CA1 neurons. *Science* 389:ead3814. 10.1126/science.adp3814 40608915

[B143] Willems J. G. P. Wadman W. J. Cappaert N. L. M. (2018). Parvalbumin interneuron mediated feedforward inhibition controls signal output in the deep layers of the perirhinal-entorhinal cortex. *Hippocampus* 28 281–296. 10.1002/hipo.22830 29341361 PMC5900730

[B144] Wilson D. I. Watanabe S. Milner H. Ainge J. A. (2013). Lateral entorhinal cortex is necessary for associative but not nonassociative recognition memory. *Hippocampus* 23 1280–1290. 10.1002/hipo.22165 23836525 PMC4030623

[B145] Witter M. P. Wouterlood F. G. Naber P. A. van Haeften T. (2006). Anatomical organization of the parahippocampal-hippocampal network. *Ann. N. Y. Acad. Sci.* 911 1–24. 10.1111/j.1749-6632.2000.tb06716.x 10911864

[B146] Wu Y. K. Miehl C. Gjorgjieva J. (2022). Regulation of circuit organization and function through inhibitory synaptic plasticity. *Trends Neurosci.* 45 884–898. 10.1016/j.tins.2022.10.006 36404455

[B147] Xiang J.-Z. Brown M. W. (1998). Differential neuronal encoding of novelty, familiarity and recency in regions of the anterior temporal lobe. *Neuropharmacology* 37 657–676. 10.1016/s0028-3908(98)00030-6 9705004

[B148] Yang C. Naya Y. (2023). Sequential involvements of the perirhinal cortex and hippocampus in the recall of item-location associative memory in macaques. *PLoS Biol.* 21:e3002145. 10.1371/journal.pbio.3002145 37289802 PMC10284415

[B149] Yanike M. Wirth S. Smith A. C. Brown E. N. Suzuki W. A. (2009). Comparison of associative learning-related signals in the macaque perirhinal cortex and hippocampus. *Cereb. Cortex* 19 1064–1078. 10.1093/cercor/bhn156 18936274 PMC2665157

[B150] Zhang X. Cao Q. Gao K. Chen C. Cheng S. Li A. et al. (2024). Multiplexed representation of others in the hippocampal CA1 subfield of female mice. *Nat. Commun.* 15:3702. 10.1038/s41467-024-47453-8 38697969 PMC11065873

[B151] Zhu Y. Wu T. Jiao Q. Chai H. Wang Y. Tian C. et al. (2025). Acute REM sleep deprivation alleviated depression- like behavior mediated by inhibiting VIP neurons in the mPFC. *Sci Adv*. 11:eadx2666. 10.1126/sciadv.adx2666 40929273 PMC12422183

[B152] Ziakopoulos Z. Brown M. W. Bashir Z. I. (2000). GABAB receptors mediate frequency-dependent depression of excitatory potentials in rat perirhinal cortex in vitro. *Eur. J. Neurosci.* 12 803–809. 10.1046/j.1460-9568.2000.00965.x 10762309

